# Local Ionic Conditions Modulate the Aggregation Propensity and Influence the Structural Polymorphism of α-Synuclein

**DOI:** 10.1021/jacs.4c13473

**Published:** 2025-04-10

**Authors:** Maria Zacharopoulou, Neeleema Seetaloo, James Ross, Amberley D. Stephens, Giuliana Fusco, Thomas M. McCoy, Wenyue Dai, Ioanna Mela, Ana Fernandez-Villegas, Anne Martel, Alexander F. Routh, Alfonso De Simone, Jonathan J. Phillips, Gabriele S. Kaminski Schierle

**Affiliations:** † Department of Chemical Engineering and Biotechnology, University of Cambridge, Philippa Fawcett Drive, Cambridge CB3 0AS, U.K.; ‡ Living Systems Institute, University of Exeter, Stocker Road, Exeter EX4 4QD, U.K.; § School of Molecular and Cellular Biology and Astbury Centre for Structural Molecular Biology, University of Leeds, Leeds LS2 9JT, U.K.; ∥ Department of Chemistry, University of Cambridge, Cambridge CB2 1EW, U.K.; ⊥ Department of Life Sciences, Imperial College London, London SW7 2AZ, U.K.; # Institut Laue Langevin, 71 Avenue des Martyrs, Grenoble CS 20156 38042, France

## Abstract

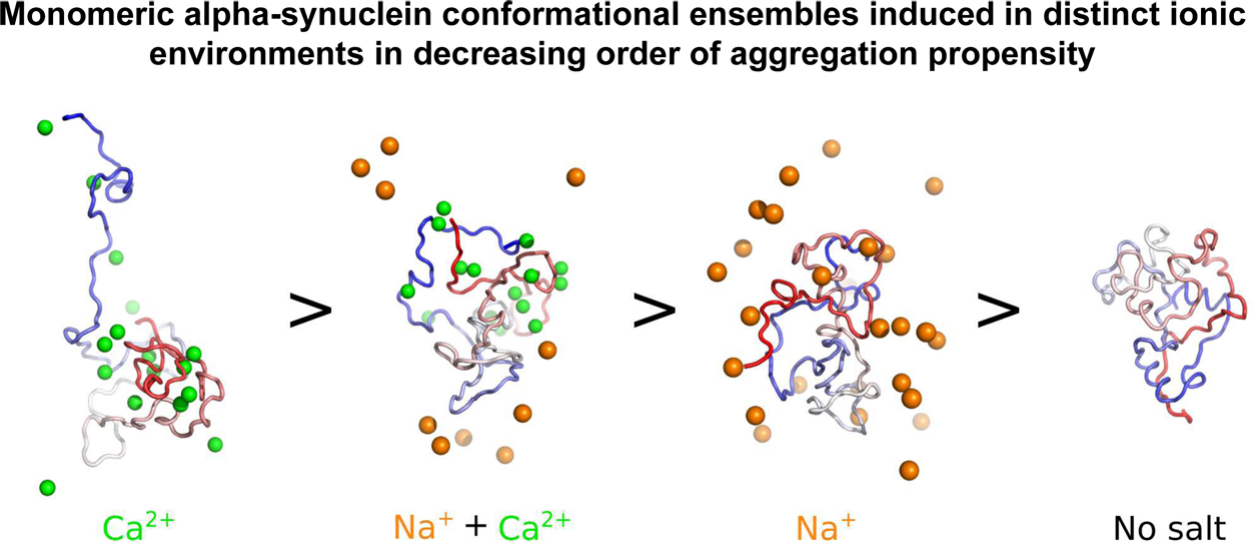

Parkinson’s disease (PD) is linked to the aggregation of the intrinsically disordered protein α-synuclein (aSyn), but the precise triggers and mechanisms driving this process remain unclear. Local environmental factors, such as ion concentrations, can influence aSyn’s conformational ensemble and its tendency to aggregate. In this study, we explore how physiologically relevant ions, mainly Ca^2+^ and Na^+^, affect aSyn aggregation, monomer structural dynamics, and fibril polymorphism. ThT fluorescence assays show that all ions speed up aggregation, with Ca^2+^ having the strongest effect. Using heteronuclear single quantum correlation nuclear magnetic resonance (^1^H–^15^N HSQC NMR) spectroscopy, we validate that Ca^2+^ binds at the C-terminus while Na^+^ interacts nonspecifically across the sequence. Small-angle neutron scattering (SANS) and hydrogen–deuterium exchange mass spectrometry (HDX-MS) show that Na^+^ leads to more extended aSyn structures, while Ca^2+^ results in moderate extension. Molecular dynamics (MD) simulations support this, showing Na^+^ increases extension between the NAC region and C-terminus, whereas Ca^2+^ biases the ensemble toward a moderately elongated structure. MD also shows that Ca^2+^ increases water persistence times in the hydration shell, indicating that aSyn aggregation propensity is due to a combination of conformational bias of the monomer and solvent mobility. Atomic force microscopy (AFM) points toward the formation of distinct fibril polymorphs under different ionic conditions, suggesting ion-induced monomer changes contribute to the diversity of fibril structures. These findings underscore the pivotal influence of the local ionic milieu in shaping the structure and aggregation propensity of aSyn, offering insights into the molecular underpinnings of PD and potential therapeutic strategies targeting aSyn dynamics.

## Introduction

Current treatments for Parkinson’s disease (PD) patients are symptomatic, as the detailed molecular mechanism leading to PD pathology remains elusive.^1,2^ Evidence points toward the aggregation of a small presynaptic protein, α-synuclein (aSyn), as it transitions from its functionally disordered form into β-sheet rich amyloid fibrils, which are present in Lewy body (LB) inclusions, one of the hallmarks of PD.^3−5^^6−14^ However, many unanswered questions remain with regard to aSyn aggregation in neurons and, in particular, when the soluble, functional form of monomeric aSyn starts to misfold into a structure that consequently facilitates aSyn aggregation. Elucidating the triggers for aSyn misfolding and the associated monomeric structures and dynamics will aid the design of effective therapeutics.

Monomeric aSyn is an intrinsically disordered 14.4 kDa protein consisting of 140 amino acid residues, which can be divided into three specific categories: a highly positively charged amphipathic N-terminus (1–60), a central hydrophobic core (61–95) known as the nonamyloid β component (NAC) region, and an acidic, C-terminal tail (96–140).^15,16^ aSyn has remarkable conformational flexibility, structural plasticity, and no unique structure, in contrast to well-folded proteins.^17^ Due to this conformational plasticity, an ensemble of different protein structures is expected to coexist, dependent on the protein’s local environment and binding partners.^18,19^ The inherent fluctuations in the structure of an unfolded protein permit residues that are separated in the primary sequence to encounter one another in space. There is increasing evidence that residual structure and intramolecular interactions exist within monomeric aSyn despite its conformational flexibility.^20−23^ These interactions are stabilized by hydrogen bonds and electrostatic and hydrophobic interactions and account for aSyn’s smaller radius of gyration compared to the predicted random-coil 140-residue protein, suggesting a partially folded structure.^24^ The partial folding of the protein has been proposed to modulate the fibrillation propensity of aSyn.^25^ It is thus important to understand whether and how bias in the conformational ensemble of aSyn, modulated by an alteration of these long-range interactions, may impact the aggregation of aSyn and the resulting fibrils.

Indeed, even though the trigger(s) that initiate the misfolding of soluble disordered aSyn into insoluble fibrils are still unknown, there is growing evidence that pathology is initiated by the disruption of the monomeric aSyn conformation.^26,27^ The conformational ensemble of aSyn is further expected to be skewed toward different conformers in different cellular compartments, where diverse microenvironments are maintained (different concentrations of ions, pH, or binding partners).^28^ The interaction of the protein with the surrounding solvent is also pertinent as ions influence the mobility of water molecules in the solvation shell of a protein. This interaction is especially relevant for intrinsically disordered proteins such as aSyn, which exhibit significantly larger solvent-accessible areas in comparison to globular proteins of similar size.^29^

There are several possible routes for aSyn to encounter different environmental conditions. The release of aSyn into the extracellular space through routes such as cell death and release of cellular contents, exocytosis, or exosome release, may lead to aSyn being exposed to high salt and high calcium concentrations.^30,31^ Subsequent uptake by endocytosis into the endosomal/lysosomal pathway would expose aSyn to a low-pH environment.^32^ Furthermore, calcium dysfunction^33^ and mitochondrial dysfunction^34^ are hallmarks of aging cells^35−37^ and may lead to alterations in the cellular environment and thus lead to aSyn structures that are trapped in a more aggregation-prone conformation.

Besides its impact on the monomer conformation, the local environment has also been shown to affect fibril polymorphism. Structural studies *in vitro* have demonstrated that the presence of salt ions can yield distinct aggregate structures, with twisted fibrils forming in the presence of salt and ribbon-like fibrils forming in the absence of salt, thereby exhibiting different levels of toxicity.^38−40^ The formation of these fibril polymorphs has also been observed and reported in the recent publications of high-resolution structures using cryoEM, where different fibril structures are formed in almost every study,^41−46^ driven by the distinct physicochemical conditions in which the fibrils are grown (*e.g.*, salt concentration, crystallization factors). Fibril polymorphism can be attributed either to the protofibril-level structure (kernel structure) or to how the protofibrils intertwine with each other to give rise to full amyloid fibrils.^44^ The existence of different kernels at the protofibril level suggests that distinct fibril polymorphs arise not only from differences in the way the two protofibrils twist around each other but also from structural characteristics that precede protofibril association. Thus, the distinct polymorphs may stem from the structural characteristics of the oligomers and/or the original monomer conformation. Recently, the structure of *ex vivo* aSyn fibrils from post-mortem tissue of patients with multiple systems atrophy (MSA), Dementia with Lewy bodies (DLB), and PD has been resolved *via* cryo-electron microscopy (EM),^47−51^ providing further evidence that different fibril polymorphs are related to different disease phenotypes.

In light of the structural plasticity of the aSyn monomer and the diversity of its aggregation products, we aim to understand how the local environment affects the conformational ensemble of aSyn, the aggregation propensity of the protein, and the structure of the fibrils formed fibrils. In our previous publications,^52,53^ we investigated the effect of the physiologically relevant Ca^2+^ ion binding on the aggregation propensity and conformational state of aSyn. By studying the aggregation kinetics of a panel of familial mutants, we uncovered distinct aggregation kinetics in response to the presence of Ca^2+^. We further established that a bias in the exposure of the aSyn monomer (especially at the N-terminus and NAC region) correlated positively with the protein’s aggregation propensity. We observed a correlation between the aSyn aggregation kinetics in distinct ionic environments, building up toward mimetics of the intracellular, extracellular, and lysosomal solvent environment.^50^ Furthermore, we showed that the solvent dynamics were an important factor affecting aSyn aggregation: ions that decreased the water mobility in the solvation shell of aSyn led to an increase in its aggregation rate.^54^ Here, we focus on the study of the conformational dynamics of aSyn for the physiologically relevant NaCl and KCl salts to elucidate the added effect of these environmental parameters on the aggregation propensity of aSyn.

In particular, we study the aggregation kinetics of aSyn under the different ionic conditions related to the intracellular and extracellular space. We investigate the monomer structural dynamics of aSyn in the same conditions by hydrogen–deuterium mass spectrometry (HDX-MS), heteronuclear single quantum coherence nuclear magnetic resonance (^1^H–^15^N HSQC NMR), and small-angle neutron scattering (SANS) and relate the differences in conformation to the protein’s aggregation behavior. Similar to our previous work,^53^ recent advances in instrument development allowed us to collect HDX-MS data at high structural and temporal resolution, with HDX labeling in the millisecond regime coupled with soft fragmentation in the gas phase (electron-transfer dissociation—ETD).^55,56^ We further model the monomeric conformation of aSyn in the same environmental ion conditions, using all-atom MD simulations, and extract structural information on the conformational ensemble of aSyn, as well as its interactions with the ions and the solvent. Finally, at the fibril level, we probe the structural polymorphism of the aSyn fibrils formed under their distinct environmental conditions *via* atomic force microscopy (AFM).

We conclude that the solution conditions assessed in this study (Na^+^, Ca^2+^, Na^+^ and Ca^2+^, K^+^) lead to distinct local conformational changes of the aSyn monomer that influence the aggregation kinetics and polymorphism of the formed fibrils. We observe that the aggregation rate of aSyn in CaCl_2_, NaCl, and KCl is increased in comparison to a “no salt” environment, with CaCl_2_ having a much stronger effect than NaCl and KCl. Both NaCl and KCl have the same effect on the aggregation kinetics; thus, we have focused our efforts on elucidating the effects of Ca^2+^ and Na^+^ on aSyn monomer structure and dynamics. We observe that Na^+^ has no clustered binding site, in contrast to Ca^2+^, which has its binding site at the C-terminus of aSyn retained in the presence of Na^+^. Both Na^+^ and Ca^2+^ ions induce an extension of the aSyn monomer structure: the addition of only Na^+^ induces the largest structural extension, followed by Na^+^ and Ca^2+^, and Ca^2+^ only. Furthermore, we show that the solvent mobility in the hydration shell of aSyn is reduced in the presence of Ca^2+^ compared to Na^+^. By correlating these observations on the monomer to the aggregation kinetics assays, we suggest that the rate of aSyn aggregation is influenced by a combination of a structural extension of the aSyn monomer, as well as by the solvent mobility in the hydration shell of the protein. This further leads to the formation of distinct fibril polymorphs in the presence of different ions, with disease-relevant, more twisted fibrils formed in the presence of Ca^2+^. Our results therefore highlight the importance of the local environment on aSyn aggregation and suggest ways for therapeutic intervention by designing molecules that (i) stabilize a more compact monomer conformation, (ii) regulate the ion household in the cell, (iii) and/or modulate the hydration shell around the protein (osmolytes) and thus increase the protein’s mobility directly to prevent aggregation.

## Results

### The Aggregation Kinetics of aSyn Is Influenced by the Local Ionic Milieu

**Figure 1:**
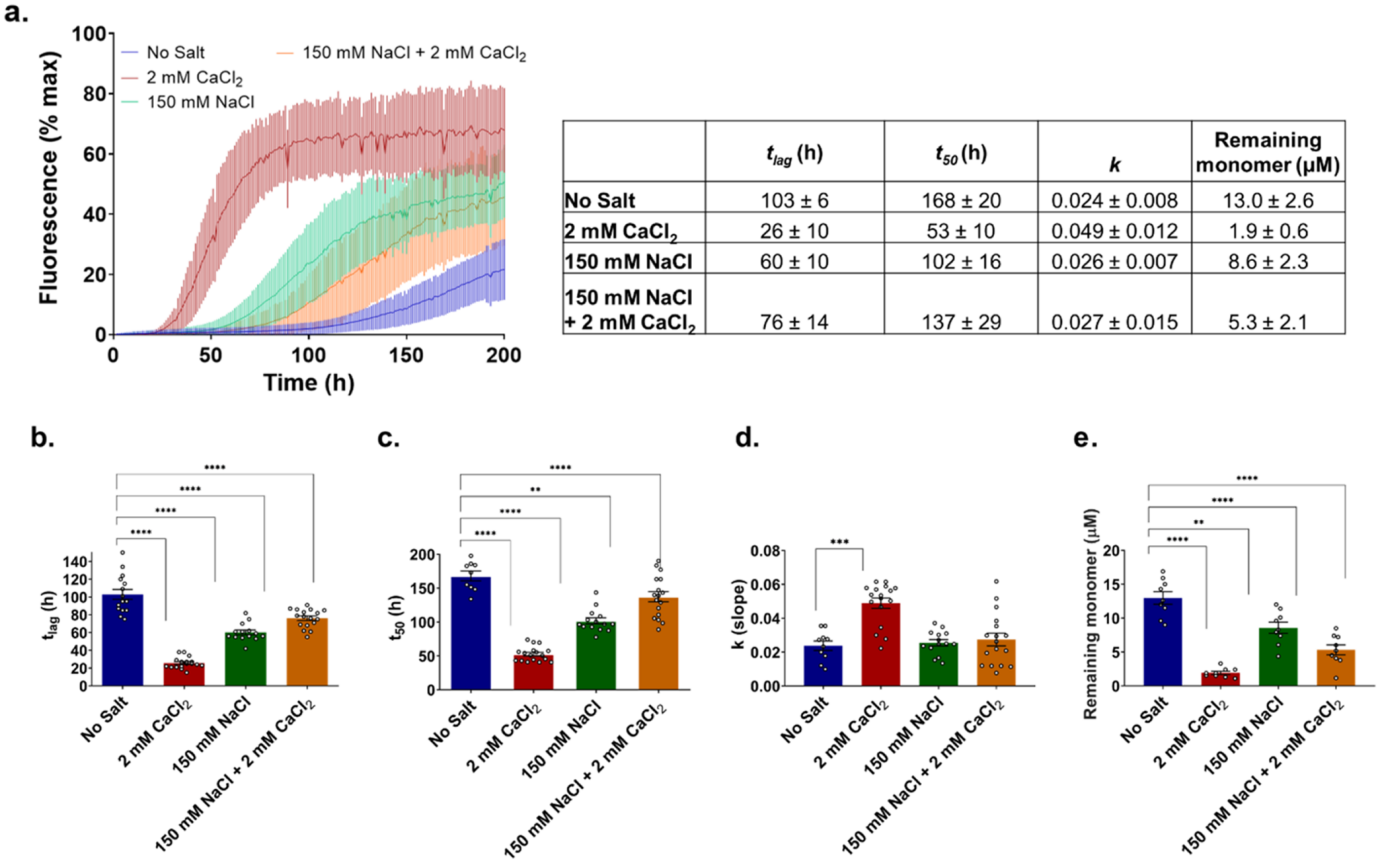
ThT aggregation kinetics of WT aSyn is increased in the presence of all ions (Na^+^, Ca^2+^), with Ca^2+^ displaying the highest increase. The aggregation kinetics of aSyn were determined by measuring ThT fluorescence intensity, which was plotted as the % of maximum fluorescence. 20 μM aSyn was incubated with 20 μM ThT in a 96-well plate with agitation at 300 rpm for 5 min before each read out every hour for 250 h. The conditions studied are aSyn in 20 mM Tris pH 7.4, with addition of 2 mM CaCl_2_, 150 mM NaCl, 150 mM NaCl, and 2 mM CaCl_2_. At least 6 replicates across three biological repeats were collected per condition. (a) Kinetic traces. The average between traces of the same condition is shown in the graph, and errors indicate 1 s.d.; (b) lag time (*t*_lag_); (c) time to reach 50% of maximum aggregation (*t*_50_); and (d) slope of the curve *k* (calculated by fitting eq 1), and the mean plus error (1 s.d.) are displayed in the graphs. (e) Remaining monomer concentration (μM) at the end of the aggregation assay was determined using SEC-HPLC, where 25 μL of sample from each well in the ThT assays was analyzed using an AdvanceBio SEC 130Å column in 20 mM Tris pH 7.4 at 0.8 mL min^–1^. An ordinary ANOVA was used to calculate statistical significance between samples, and significant differences are reported on the graph with an asterisk *. The averages, standard deviations, and p values are presented in the SI.

We have previously investigated the aggregation and structure of aSyn monomer in complex ionic environments, which mimic different cellular compartments, showing increased aggregation at low pH and high Ca^2+^ concentrations.^52,53^ Here, we want to further probe the mechanisms of aggregation kinetics twinned with monomer structural assessment by studying a more simplistic environment that is easier to model with MD simulations. We first investigate how the major ion components of the local physiological environment, namely, Na^+^, K^+^, and Ca^2+^ ions, influence the aggregation rate of aSyn, building up to *in vitro* ion mimetics of the intracellular and extracellular environment. To do so, we first employ a ThT fluorescence-based assay, widely used in the amyloid field.^57^ Briefly, the ThT molecule fluoresces when bound to rich fibrillar β-sheet structures, such as those found in amyloid fibrils, and thus, the assay provides us with a tool to measure aSyn aggregation rates. The aggregation kinetics typically follow a sigmoidal curve. Aggregation kinetics are fitted, and three parameters are extracted, namely, *t*_lag_ (the time spent in the lag phase, corresponding to nucleation), *k* (the slope of the exponential phase, corresponding to fibril elongation), and *t*_50_ (the midpoint of the exponential phase, also corresponding to elongation). We probe the aSyn aggregation rates for five separate conditions: aSyn in Tris buffer only, pH 7.4, aSyn in 2 mM CaCl_2_, and aSyn in 150 mM KCl (corresponding to the intracellular ionic environment, Figure S2), aSyn with 150 mM NaCl, and aSyn with 150 mM NaCl and 2 mM CaCl_2_ buffer solutions (corresponding to the extracellular ionic environment) (Figure 1a–d). We also measured the remaining monomer concentration at the end of the ThT-based assays as an orthogonal method to determine the extent of aSyn aggregation (Figure 1e). Distinct aggregation kinetics can be identified for each condition, indicating the different effects ions have on the aggregation propensity of aSyn.

All ions enhance the aggregation rate of aSyn, as evident when comparing the (Tris) buffer only condition “No Salt” to all other conditions. The Na^+^ and K^+^ ions both increase the aggregation rate, halving the *t*_lag_ and *t*_50_. However, the most distinct effect on aSyn aggregation is observed in the 2 mM CaCl_2_ condition, with a clear decrease in the aSyn nucleation time and an increase in the slope of the exponential elongation phase. The divalent Ca^2+^ ion speeds up the aggregation rate of aSyn to a much greater extent than the monovalent Na^+^ and K^+^ ions, even though its concentration is 75 times lower (2 *vs* 150 mM). In the presence of both Ca^2+^ and Na^+^ ions, the aggregation rate of aSyn is comparable to the rate in the presence of only Na^+^. These observations, in combination with the predicted absence of a structured binding site for Na^+^ and K^+^ ions,^58^ indicate that the different ionic conditions affect the aSyn kinetics differently. We have hypothesized that the binding of Ca^2+^ at the C-terminus modulates the release of long-range interactions of the aSyn monomer, while the Na^+^ and K^+^ ions have a nonspecific, electrostatic effect on the monomer.^58^ To determine whether our observations from the kinetic assays can be attributed to structural changes in the aSyn monomer, further studies are required. We thus decided to analyze the aSyn monomer structure and dynamics with a panel of biophysical techniques: ^1^H–^15^N HSQC NMR, the cutting-edge millisecond HDX-MS, and SANS. In particular, we focus our efforts on disentangling the distinct effects of Ca^2+^ and Na^+^ ions on aSyn.

### The Conformational Ensemble of Monomeric aSyn Is Influenced to Various Degrees by the Presence of Different Ions

**Figure 2:**
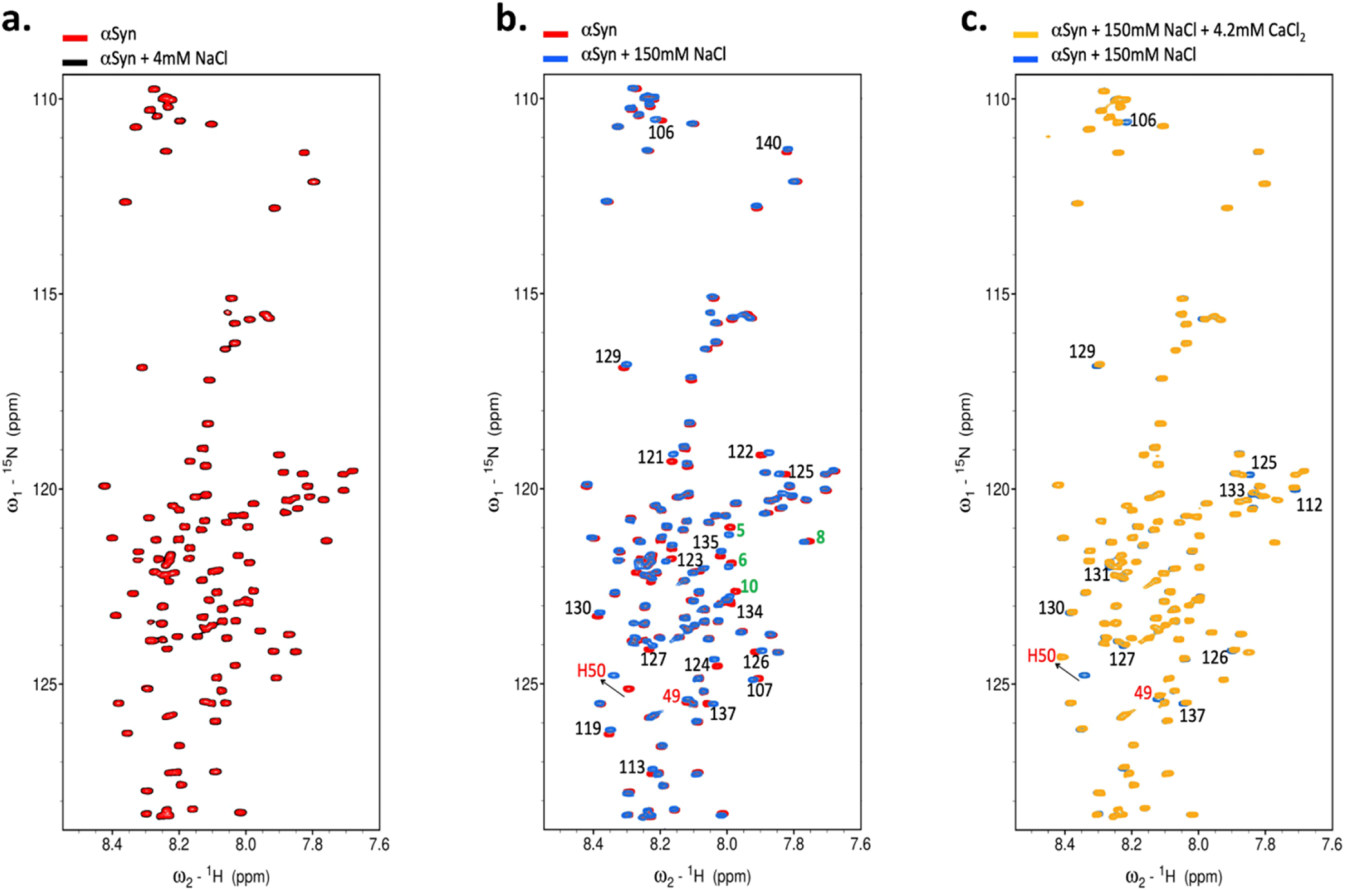
^1^H–^15^N HSQC NMR shows CSPs across the sequence of monomeric aSyn in the presence of NaCl and specific residue interactions at the C-terminus in the presence of Ca^2+^. (a) We do not observe chemical shift perturbations (CSPs) in the ^1^H–^15^N resonances of aSyn amide backbone upon the addition of 4 mM NaCl. (b) Addition of 150 mM NaCl causes CSPs primarily at the C-terminus (*e.g.*, residues 121, 122, 123, *etc.*, labeled black) and at the N-terminus (*e.g.*, residues 5, 6, 8, 10, *etc.*, labeled green). These peak motions are attributed to the weakening of the electrostatic interactions between the N- and C-terminal regions, as well as to possible rearrangements within the C-terminal region (where repulsion between negative charges diminishes due to the higher ionic strength). We also observed CSPs for H50 and the neighboring residue 49 (labeled red). These CSPs are attributed to alterations in the p*K*_a_ (from 6.8 to 6.5) due to the change in salt concentrations (from 4–150 mM)^59^. (c) Comparison of aSyn in 150 mM NaCl and 150 mM NaCl + 4.2 mM CaCl_2_ shows CSPs at C-terminal residues (arrows with assigned amino acid residues) likely associated with direct binding to Ca^2+^. aSyn concentration was fixed at 200 μM across experiments. The ΔCS are represented as a histogram in the Supporting Information (Figure S9).

Previously,^52^ we have studied the binding of Ca^2+^ ions to aSyn *via* HSQC NMR. Here, we extend this study to probe the aSyn monomer structure in the presence of 4 mM NaCl, 150 mM NaCl, and 150 mM NaCl with 4.2 mM CaCl_2_ (Figure 2a–c).

We previously observed chemical shift perturbations (CSPs) at the C-terminus upon calcium binding (residues 104, 107, 112, 119, 123, 124, 126, 127, 129, 130, 135, 136, and 137), which correlate with the expected calcium-binding site. In contrast, 4 mM NaCl (Figure 2a) does not induce any CSPs on the aSyn monomer spectrum, showing that the identified Ca^2+^ effect at the C-terminus is specific to the ion and can be attributed to a localized binding site. At 150 mM NaCl (Figure 2b), CSPs can be observed at C-terminal residues (labeled in black, 106, 107, 113, 119, 124, 127, 129, 130, 134, 135, 137, 140) and at certain N-terminal residues (labeled in green, 5, 6, 8, 10). These peak motions can be attributed to a charge effect, a weakening of the intramolecular electrostatic interactions between N- and C-terminal regions, as well as to possible rearrangements within the C-terminal region, where repulsion between negative charges diminishes due to the high ionic strength. Intriguingly, we also observe peak changes at H50 and the neighboring residue 49 (labeled red) as a result of the alteration in p*K*_a_ (from 6.8 to 6.5) due to the change in salt concentrations (from 4–150 mM).^59^ In the presence of 150 mM NaCl and 4.2 mM CaCl_2_ (Figure 2c), the CSPs previously recorded at the C-terminus (106, 112, 125, 126, 127, 129, 130, 131, 133, and 137) are still identifiable with respect to the 150 mM NaCl spectrum, proving that the binding site is retained in the presence of NaCl, and thus Ca^2+^ binding still occurs at the C-terminus even in the presence of 150 mM NaCl.

**Figure 3:**
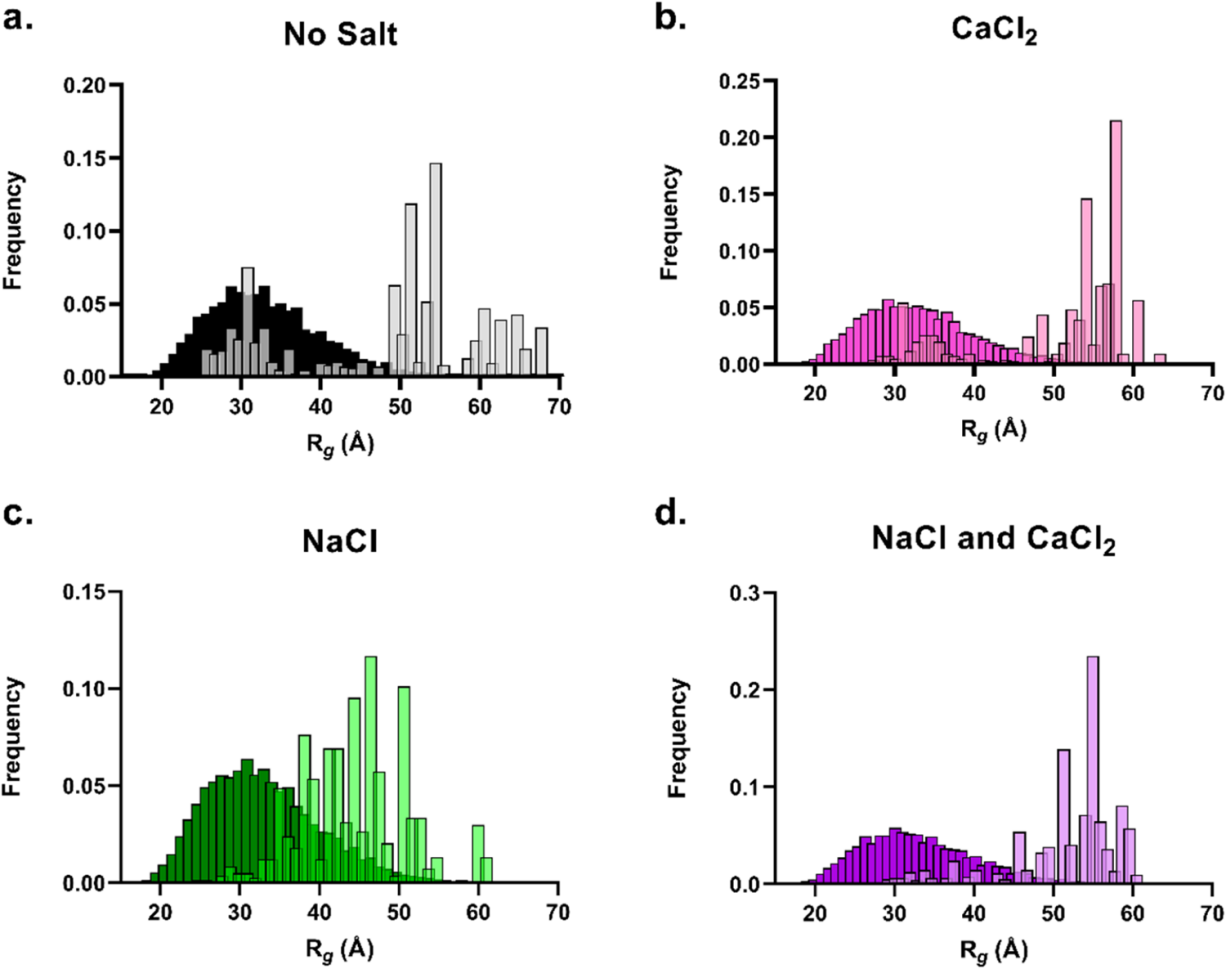
Small-angle neutron scattering data show that Na^+^ induces an average extension of the conformation population of aSyn, whereas Ca^2+^ promotes a more extended conformation population. A pool of 10000 independent models (dark bars in each graph) based upon sequence and structural information (*i.e.*, no defined structure for an IDP) is generated. The predicted scattering intensity from the models is compared to the experimental data, and the 50 models of the best fit to the experimental data are selected as the most accurate representations (light bars in each graph). (a) aSyn in the “no salt” tris buffer samples has the widest distribution of *R*_g_ (gray bars). (b) aSyn in the CaCl_2_ buffer samples show two main conformational spaces: a more compact structure ∼30 Å and a larger structure ∼60 Å (pink bars). (c) aSyn in NaCl samples show a more averaged size of conformational space ∼40–50 Å (green bars). (d) aSyn in the presence of both NaCl and CaCl_2_ has larger *R*_g_, mostly between 50 and 60 Å (purple bars). The aSyn concentration was 20 μM, and the different ionic conditions tested were 20 mM Tris in the presence of 150 mM NaCl, 2 mM CaCl_2_, or 150 mM NaCl + 2 mM CaCl_2_.

We next employ SANS to characterize conformational changes and radii of gyration of aSyn monomers in different ionic conditions. Monomeric aSyn is measured in buffer only, in the presence of 2 mM CaCl_2_, 150 mM NaCl, and 150 mM NaCl mixed with 2 mM CaCl_2_, and the radius of gyration of the protein is calculated for each condition *via* an ensemble optimization method (EOM) (Figure 3). In the absence of salts (“no salt condition”), aSyn samples display the widest distribution of *R*_g_. In the presence of Ca^2+^, aSyn samples show two main conformational spaces: a more compact structure of ∼30 Å and a larger structure of ∼60 Å. aSyn in NaCl exhibits a broader conformational space of ∼40–50 Å, while aSyn in the presence of both Na^+^ and Ca^2+^ has a larger *R*_g_ between 50 and 60 Å. The presence of Ca^2+^, in the absence and presence of Na^+^, shifts the conformation to favor more extended structures compared to the “no salt” conditions, normalizing the ensemble of monomers toward a radius of gyration between 50 and 60 Å. These results suggest that these cations may facilitate desolvating aSyn in water, thus promoting aggregation of the protein.

Having identified the differences between binding the of Ca^2+^ and Na^+^ on aSyn *via* NMR and the changes in the overall shape of the protein *via* SANS, we set out to capture the more localized and faster-changing protein dynamics of the aSyn monomer. We thus use millisecond HDX-MS with soft fragmentation *via* electron-transfer dissociation (ETD), a technique that can be used to measure the dynamics of intrinsically disordered proteins at a very high temporal (subsecond mixing) and structural (ETD fragmentation) level.

**Figure 4:**
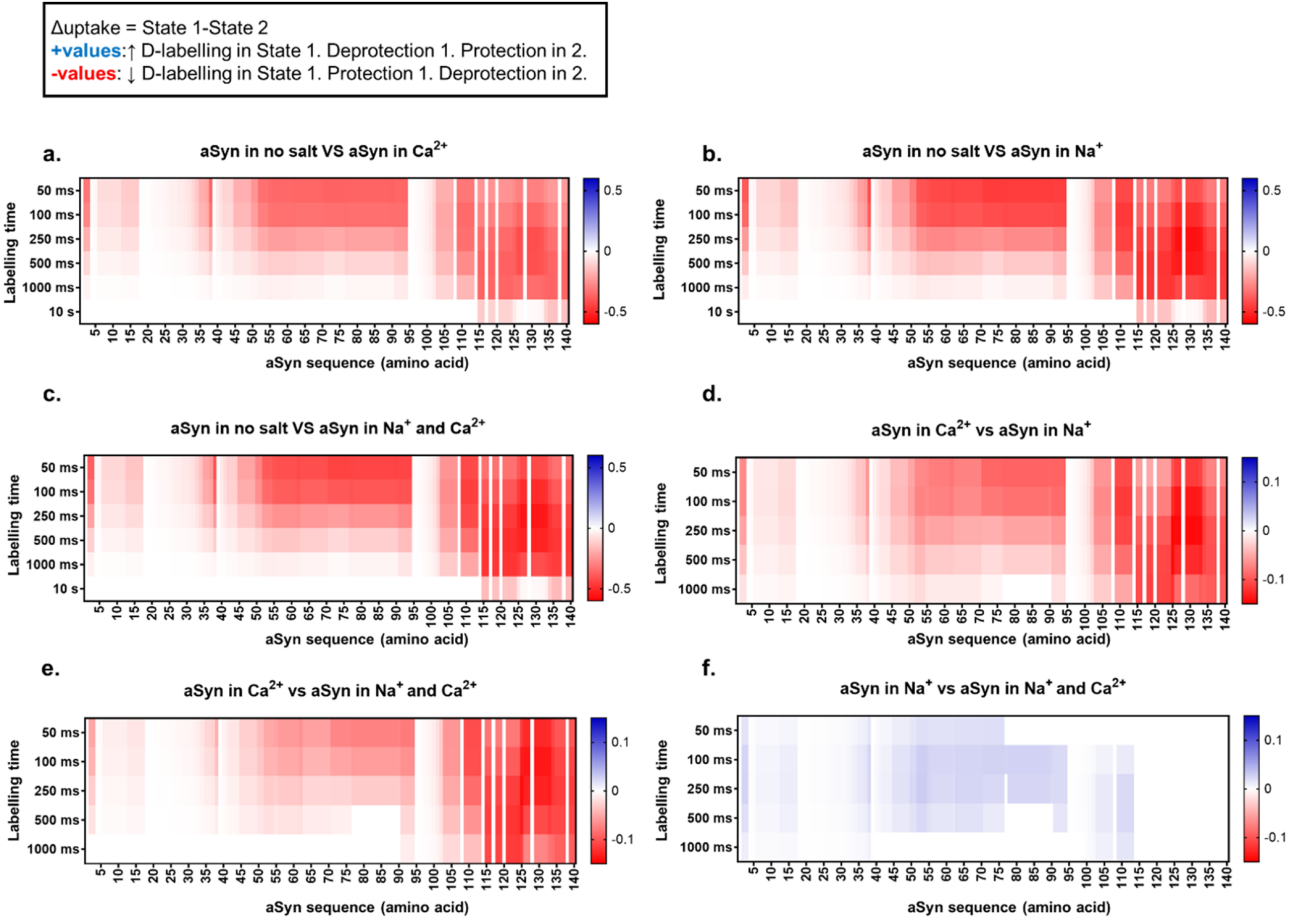
HDX-MS reveals that all ions induce a deprotection of monomeric aSyn, with Na^+^ having the strongest effect. Heatmaps showing significant differences (nonwhite) in deuterium uptake per time point during an on-exchange reaction between STATE 1 and STATE 2 (*e.g.*, aSyn in no salt *vs* aSyn with Ca^2+^). The conditions studied are 5 μM aSyn in 20 mM Tris pH 7.4 in the presence of 2 mM CaCl_2_, 150 mM NaCl, 150 mM NaCl, and 2 mM CaCl_2_. *X*-axis: protein sequence, *y*-axis: HDX labeling time point (50 ms–10 s). Positive values are in red and represent decreased uptake in STATE 2, whereas negative values are in blue and represent increased uptake in STATE 2. Increased uptake indicates more solvent exposure and/or less participation in stable hydrogen-bonding networks. (a) Monomeric aSyn is more protected when no salt is present compared to the addition of Ca^2+^. (b) the addition of Na^+^. (c) Addition of Na^+^ + Ca^2+^. (d) Monomeric aSyn in Ca^2+^ is more protected compared to in Na^+^. (e) aSyn in Ca^2+^ is more protected than in Na^+^ and Ca^2+^, and (f) aSyn in Na^+^ is less protected than in Na^+^ and Ca^2+^. Data analysis was performed in DynamX (Waters), and hybrid significance testing was performed using Welch’s *t* test (*p*-value of 0.05) and global significance thresholding.

The aSyn monomer is incubated in deuterated buffer (D_2_O) for time points ranging from 50 ms to 30 s in different conditions (Buffer only, 2 mM CaCl_2_, 150 mM NaCl, 150 mM NaCl, and 2 mM CaCl_2_). It should be noted that the HDX rate is itself affected by the ionic strength of the buffer. For this reason, uncorrected deuterium uptake consists of the sum of the contributions of the ions to the HDX chemical exchange rate and the changes in HDX due to differences in the conformation of the protein (which we set out to probe). The data are thus corrected for the contribution of environmental conditions to the HDX chemical exchange rate using the model disordered peptide, bradykinin, in the exact same buffer conditions as aSyn, as previously described.^53,60^ Therefore, we are able to make direct comparisons between the effect of the ions on the aSyn conformational ensemble. The HDX-MS data are plotted as the difference in deuterium uptake between protein states for each labeling time point (50 ms to 30 s) across the protein sequence (Figure 4). This results in a deuterium uptake heatmap, in which positive values (blue) indicate that protein state 1 is deprotected compared to protein state 2, and negative values (red) indicate that state 1 is protected compared to state 2.

When comparing the deuterium uptake of the “No salt buffer only” condition to all other conditions (aSyn + Ca^2+^, aSyn + Na^+^, aSyn + Ca^2+^ + Na^+^), aSyn is in its most protected/compacted conformation in the “No salt buffer only” condition, indicating that all tested ions cause deprotection (Figure 4a–c). This deprotection is most pronounced at the NAC region residues 60–90 and residues 120–135 of the C-terminus. A binary comparison of aSyn deuterium uptake in the presence of Ca^2+^*versus* in the presence of Na^+^ (Figure 4d) shows that aSyn in the Na^+^ state is more deprotected/exposed, particularly at residues 60–90, 109–112, 120–125, and 130–135. A similar conclusion can be drawn when comparing aSyn in the Ca^2+^ condition *versus* the Na^+^ + Ca^2+^ condition (Figure 4e). The Na^+^ + Ca^2+^ state is more protected/compact than the Na^+^ state, particularly at residues 109–112, 120–125, and 130–135 of the C-terminus and residues 60–90 of the NAC region. To summarize, aSyn is in its most protected state when there are no salt ions present, is moderately deprotected in the presence of Ca^2+^, more deprotected when Na^+^ and Ca^2+^ are added, and most deprotected in Na^+^ only (deuterium uptake: Na^+^ > Na^+^ + Ca^2+^ > Ca^2+^ > No salt).

Overall, the information from the biophysical studies of the aSyn monomer structure in response to the different ions can be summarized in a model as follows: aSyn in buffer only (no salt, no Ca^2+^) inhabits a compact conformational ensemble. The addition of Na^+^ biases the conformational ensemble to more extended, deprotected structures (higher *R*_g_ at ∼40–50 Å, higher deuterium uptake), with no localized binding site (CSPs across the sequence at the N-terminus and C-terminus). The addition of Ca^2+^ leads to a more moderate conformational extension throughout the sequence (higher deuterium uptake, with two subpopulations at *R*_g_ at ∼30 and ∼60 Å), with localized perturbations at the C-terminus (CSPs at C-terminal residues), at the expected Ca^2+^ binding site. In the presence of both ions, the calcium-binding site is retained (CSPs detected at the C-terminus), and the conformational ensemble is biased toward more extended structures, which are less extended than the Na^+^ only condition, but more extended than the Ca^2+^ only condition.

To further validate our interpretation of the biophysical data and to relate the monomer observations back to the aggregation rate of aSyn, we used molecular dynamics (MD) simulations to model the protein in the distinct local environments it encounters, to compare modeled aSyn monomeric to experimentally observed structured using ensemble techniques but, importantly, also to study the influence of the hydration shell of aSyn in the presence of the different ions.

### Modeling the aSyn Conformation Ensemble in Distinct Ion Environments Confirms Structural Changes Observed by Other Biophysical Techniques and Reveals That Ca^2+^ Significantly Slows down Water Mobility in the Hydration Shell of aSyn

We used MD simulations to model aSyn monomer structures under conditions with minimum salt ions, 150 mM Na^+^, 34 Ca^2+^ ions, and a combination of 150 mM Na^+^ and 34 Ca^2+^ ions. In each case, the system was neutralized with Cl^–^ ions. We have chosen 34 Ca^2+^ ions as aSyn acts as a Ca^2+^ scavenger and also because we want enough Ca^2+^ ions to saturate the binding site while allowing for approximately 10 mM CaCl_2_ left in solution. We extract monomers from PDB structures 2n0a (resolved by ssNMR), 8ads (resolved by cryoEM), and 8adw (resolved by cryoEM) as starting conformations in the simulations. Missing residues in 8a9l and 8ads are replaced by rigid body splicing of residues present in 2n0a using PyMOL. The simulations are run for over 1.5 μs at 303.15 K, and we observe the conformational changes of the monomer throughout the simulation window as well as the ion contacts to the protein. Further to that, we observe the water molecules in the hydration shell of the protein in fine time-point simulations, where each solvent molecule’s total displacement is measured every 5 ps over a period of 3 ns, allowing the study of the solvent behavior around the protein in the different environmental conditions.

**Figure 5:**
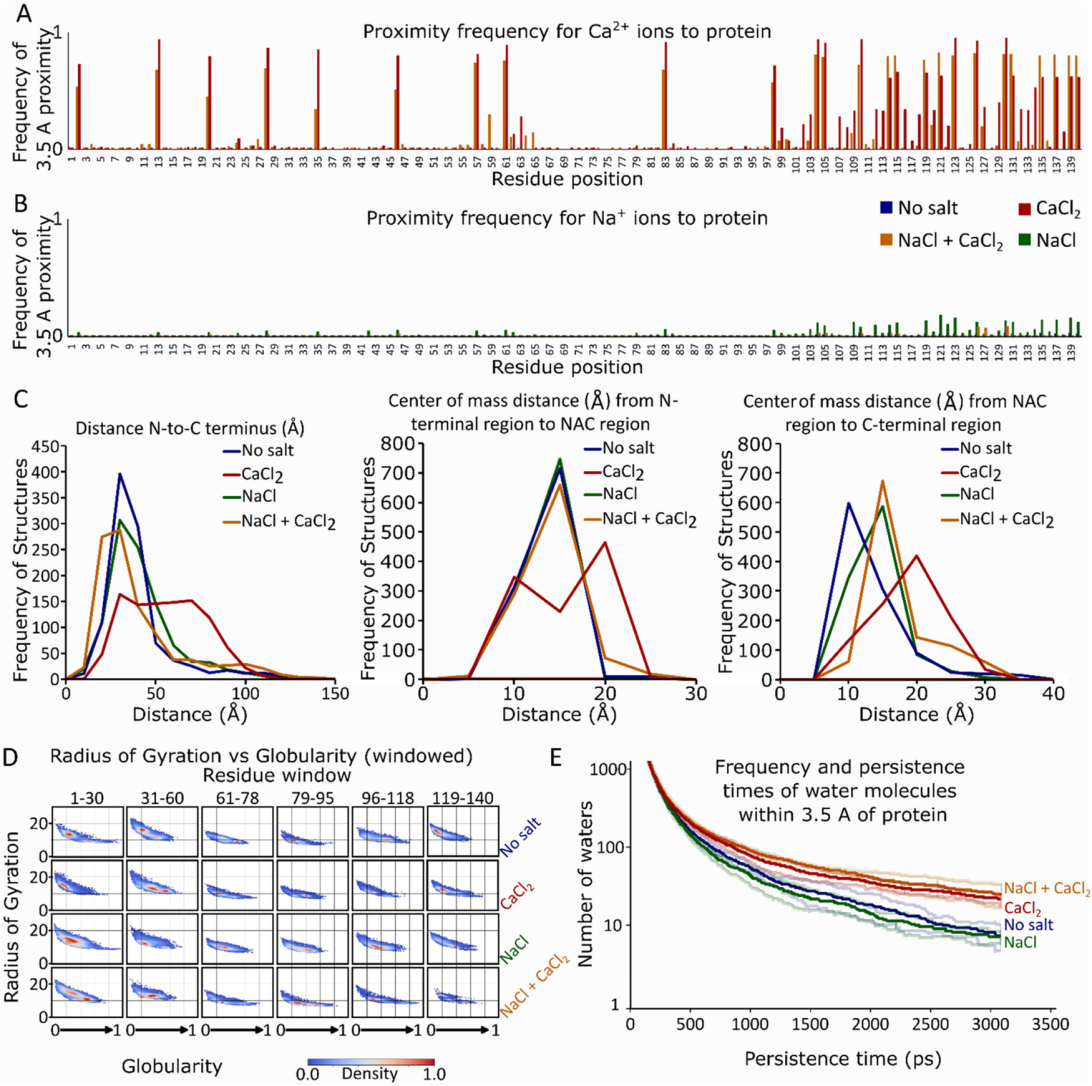
MD Simulations on monomeric aSyn in different ionic conditions confirm experimental results and reveal that Ca^2+^ ions have a higher aSyn persistence time compared to Na^+^ and that Ca^2+^ reduces solvent mobility around aSyn. (a, b) Proximity frequency of Na^+^ and Ca^2+^ ions to the aSyn monomer. (c–e) Distance of the N-terminus to the C-terminus, N-terminus to the NAC region, and NAC region to the C-terminus in different ionic conditions, indicating conformational changes. (f) Radius of gyration (*R*_g_) plotted against protein globularity across the sequence (six residue windows) in different ionic conditions. (g) Persistence time of water around aSyn in different ionic conditions. The intention of the simulations was to restrict, satiate, and saturate the calcium-binding sites. As such, simulations were carried out under the following conditions: “No Salt” contained the minimum ions required to equilibrate the system (10 Na^+^), “Unsaturated CaCl_2_“ (20 Ca^2+^ ions), “CaCl_2_” (34 Ca^2+^ ions), “NaCl and CaCl_2_” (150 mM Na^+^ and 34 Ca^2+^ ions), “NaCl” (150 mM Na^+^), and “Saturated CaCl_2_” (150 mM Ca^2+^), in each instance Cl^–^ ions were used to equilibrate the charge. All data are averaged among each starting simulation model (PDB structures 2N0A, 8A9L, and 8ADS).

By observing the Na^+^ and Ca^2+^ persistence times on the aSyn monomer (Figure 5a,b), we can infer that the binding of Ca^2+^ to aSyn is tighter, while Na^+^ ions have only transient interactions with the protein. This strengthens our previous experimental observations that indicate that the Na^+^ ions are simply adducts on the aSyn monomer. To disentangle the conformational dynamics of the protein under different conditions, we plot the distances sampled between different areas of the protein in response to the added ions (Figure 5c–e). Overall, the addition of Na^+^ pushes the distribution toward slightly more elongated structures, particularly increasing the distance between the NAC region and the C-terminus. The Ca^2+^ ions also bias the conformational ensemble toward more elongated structures, increasing both the distance between the N-terminus and the NAC region as well as the distance between the NAC region and the C-terminus.

To gain further information on the shape of the protein and its compaction/extension, we calculate and plot the radius of gyration, *R*_g_, *versus* the globularity of the protein for each of its primary sequence regions (N-terminus, early 1–30 and late 31–60; NAC region, early 61–78 and late 79–95; and C-terminus early 96–118 and late 119–140) (Figure 5d). Overall, when comparing the aSyn monomer (full length) in the “No salt” condition to the ion conditions, we infer that the Na^+^ ions do not drastically alter the conformational ensemble, whereas the Ca^2+^ ions shift the population toward more extended (higher *R*_g_ values) and less globular (lower globularity values) structures, and a mix of the two ions slightly shifts the population toward more globular structures (Figure 5d). More specific information can be extracted for different areas of the protein: Na^+^ pushes toward more extended and less globular conformations at the early N-terminus (1–30) and toward more globular and less extended conformations at the NAC region and the C-terminus. The Ca^2+^ ions cause an extension and thus a decrease in globularity at the N-terminus (particularly at the early N-terminus, 1–30), have no significant effects at the NAC region, and cause a concurrent decrease in globularity at the early C-terminus as well as an increase in globularity at the late C-terminus, perhaps indicating a structural rearrangement near the binding site. A mix of the two ions pushes toward an extension of the N-terminus and an increase in globularity at the NAC region and the C-terminus. Overall, we can see that both ions induce extension at the N-terminus, while most of the differences between Ca^2+^ and Na^+^ can be traced to the Ca^2+^ binding site at the C-terminus. These observations correlate well with our experimental biophysical data (NMR, HDX-MS, and SANS) on the aSyn monomer.

To further explain our observations in the bulk aggregation kinetics experiments, we next focus on the hydration of the protein in different conditions by assessing the persistence time of water molecules around the aSyn monomer (Figure 5e). The highest values of water persistence are observed in the presence of Ca^2+^, while the addition of Na^+^ does not have such a dramatic effect on the water mobility in the solvation shell. We observe that the presence of Ca^2+^ slows down the solvent in the hydration shell of the aSyn monomer, which correlates well with the increased aggregation propensity observed in the aSyn aggregation kinetics experiments.

### The Presence of Different Ions Impacts Fibril Morphology

To investigate whether the detected differences in monomer conformation correlate with the formation of distinct fibril polymorphs, the structure of the fibrils at the end of the kinetic assays is probed *via* atomic force microscopy (AFM). In order to quantitatively assess the morphological features of the various species, measurements of fibril height (*h*) and periodicity (*p*) are acquired (Supporting Figure S15a–d).

In all conditions, a percentage of the total fibril population is nonperiodic and contains rod-like fibrils, with *h* = 7.5 ± 1.7 nm, which we term polymorph p1. In the “No salt” condition, two more fibril polymorphs are detected: polymorph p2a (*h* = 8.6 ± 1.4 nm and *p* = 422 ± 18 nm), with a height approximately corresponding to aSyn protofibrils, and polymorph p2b (*h* = 13.8 ± 1.4 nm and *p* = 413 ± 33 nm), with a height corresponding to aSyn mature fibrils. Considering that the periodicity of the p2b fibril population (p2b) is approximately equal to the periodicity of the second population (p2a), it is very likely that the shorter fibrils of the p2a polymorph intertwine to form the mature tall fibrils of p2b.

The addition of Ca^2+^ biases the formation of more periodic twisted fibrils. Polymorph p1 can still be seen in the sample, but the second, dominant population (relative abundance 75%) of fibrils is polymorph p3a (*h* = 8.7 ± 0.7 nm and *p* = 96 ± 15 nm). Some fibrils with the same periodicity (111 ± 5 nm) but increased height (13.5 ± 0.5 nm) are also detected and termed “p3b.” This again indicates that the fibrils p3a and p3b, which share the same periodicity, are the protofibrils and full fibrils of the same species. In the presence of Na^+^, aSyn forms fibrils that can be classified into four populations: the p2a and p2b polymorphs with the lower periodicity, previously seen in the “No salt” sample, some p3a fibrils with higher periodicity, as well as a number of nonperiodical fibrils, p1. In the presence of Na^+^ and Ca^2+^, aSyn primarily forms polymorphs p3a and p3b (∼70% relative abundance), while some nonperiodical fibrils, p1, can also be detected (∼30% relative abundance). The “Na^+^ + Ca^2+^” condition is therefore very similar to the “Ca^2+^” condition, as the main fibril population arising in both cases is the more twisted periodical species of the polymorph p3a.

## Discussion

Studying the conformational dynamics of the α-synuclein monomer is fundamental for elucidating the molecular mechanisms underlying its aggregation but also to pave the way for the development of effective therapies. By focusing on the monomer dynamics, we can identify transient structural motifs or regions with a propensity to adopt certain secondary structures which are ‘on path’ to oligomerization and aggregation. In the present study, we interrogate the effect of physiologically relevant ions, primarily Ca^2+^ and Na^+^, on the conformational ensemble of the aSyn monomer and thus focus on the earliest stages of the aggregation process. We link our information on the monomer structure to its aggregation propensity and the resulting fibril polymorphism. We have shown that the Ca^2+^ and Na^+^ ions impact the monomeric structure in distinct ways.

We have used a panel of biophysical techniques, each of which provides a different nature of information, to probe the effect of the ions on aSyn. NMR generates high-resolution data on specific atomic interactions and local structural changes but cannot distinguish between the extension or compaction of the monomer structure. SANS provides low-resolution, ensemble-averaged data about the overall shape and size of the protein in solution, offering insights into global conformational changes but might miss subtle local structural changes. HDX-MS provides information about the dynamics and flexibility of different regions of the protein and highlights regions with varying degrees of protection, indicating structural stability and dynamics, but has a lower spatial resolution compared to NMR. The orthogonality of these biophysical techniques, in addition to MD simulations, has allowed us to build toward a simple structural model of the effect of ions on the conformational ensemble.

**Figure 6:**
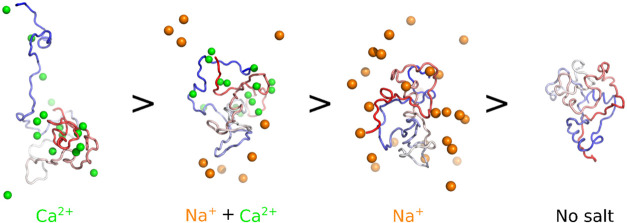
Proposed model for the aSyn conformational ensembles induced in distinct ionic environments, as informed by a combination of biophysical techniques and MD simulations, in reducing order of aggregation propensity. aSyn (N-terminus—red, NAC region—white, C-terminus—blue) in the presence of Na^+^ induces a moderate effect of extension on the aSyn monomer structure and does not contain a defined structured binding site on aSyn. In contrast, Ca^2+^ has a localized binding site at the C-terminus, which is retained in the presence of Na^+^, and binding of Ca^2+^ on the monomer structure leads to an extension of the N-terminus and the NAC region. In the absence of salt ions, the aSyn monomer samples a mostly compact conformation. Representative simulation poses are displayed for each ionic condition (corresponding to starting structure 8a9l at frame 3000 (1.5 μs)).

To summarize, the Na^+^ ion shows a general effect of extension on the aSyn monomer structure (HDX-MS and SANS) and no structured binding site. MD simulations support these observations by demonstrating that Na^+^ facilitates an extended conformation, especially in the NAC region and the C-terminus. In contrast, Ca^2+^ has a localized binding site at the C-terminus, which is retained in the presence of Na^+^, and binding of Ca^2+^ to the monomer leads to a moderate extension of the N-terminus and NAC region (HDX-MS, SANS, MD). A schematic of the aSyn monomer under each condition can be found in Figure 6.

It must be noted that we observe a high persistence time of Ca^2+^ ions at the N-terminus (*e.g.*, residues 2, 13, 20, 28) in the MD simulations, while in the NMR experiments, the CSPs upon Ca^2+^ addition are localized at the C-terminus. We attribute this difference in information to the length of the simulations; aSyn is exposed to the ions for 1.5 μs, allowing the formation of “initial” ensembles, which may not represent the structural distributions in equilibrated ensembles. While the simulations may not have captured a “final conformational state,” they still highlight interesting ion and water effects even during the formation of these early conformers.

With regard to linking the conformational ensemble to the protein’s aggregation kinetics, we observe that an extension of the monomeric structure aids to increase the protein’s aggregation propensity (“no salt condition” compared to the ion conditions). This agrees with our previous observations^52,53^ and multiple reports in the literature highlighting the effect of monomeric structure on aggregation kinetics.^52,62−66^ In our previous publication, we have shown that the local ionic environment influences shifts in the aSyn conformational ensemble that are correlated with the aggregation kinetics, while here, we have attempted a more sophisticated deconvolution of the individual ion contributions. The N-terminus is highlighted here as an area of particular importance. A recent study^67^ has shown that deletion or substitution of the residues 36–42 at the N-terminus prevents aggregation, while three N-terminal truncations (deletions of 1–13, 1–35, and 1–40 residues at the N-terminus) have been shown to modulate both the aggregation kinetics, resulting in different fibril morphologies.^68^

We propose that simply a bias toward more extended structures is not sufficient to explain the increase in aggregation propensity of the protein. That is because the most extended structures are observed in the presence of Na^+^, while the fastest aggregation kinetics are observed in the presence of Ca^2+^. We and others have shown that water plays an important role in protein aggregation,^54,69,70^ and decreased water mobility in the solvation shell correlates with an increased aSyn aggregation propensity.^54^ In this study, we observe increased water persistence times in the hydration shell in the presence of Ca^2+^ compared to Na^+^*via* MD simulations. This correlates well with the aSyn aggregation kinetics in the presence of both ions (Na^+^ and Ca^2+^), which are intermediate compared to the kinetics in the presence of the individual ions, as Na^+^ seems to balance the effect of Ca^2+^. With regard to the mechanism of solvation, we propose that the binding of Ca^2+^ ions can stabilize the rotameric conformations of the acidic residues involved in the binding interaction, which may alter the dynamics of the hydration shell. Specifically, Ca^2+^ binding neutralizes the local electrostatic environment, and as a divalent ion, it facilitates additional interactions with another acidic residue. This binding leads to localized stabilization of the side-chain dynamics, reducing their hydrogen-bonding potential. Combined with the electrostatic neutralization, this makes the protein surface less interactive with water molecules, causing water in the surrounding area to hydrogen bond more strongly with itself rather than with the protein. This creates a more ordered and stable hydration cage, reducing water exchange with the bulk solvent. We, overall, suggest that the aggregation kinetics of monomeric aSyn is increased by a combination of conformational bias toward more extended structures, particularly at the N-terminus, together with a decreased water mobility in the solvation shell of the protein. Future experiments to further corroborate this claim will need to focus on investigating the mobility of the protein in the different ionic environments *via*^1^H–^15^N HSQC NMR spectroscopy and terahertz (THz) spectroscopy.^54^

Regarding fibril polymorphism, AFM reveals a bias in the fibril morphologies formed under different ionic conditions. This finding suggests that the conformational biases induced by specific ions in the monomer state can lead to the formation of diverse fibril structures, which may correlate with the heterogeneous nature of Lewy bodies in PD. Further experiments in the future could include a more detailed characterization of the formed fibrils in each condition, for example, *via* the use of CryoEM, which allows probing the structure of the fibril core, and is a nonaveraging technique.^61^ A difference in fibril polymorph toxicity has been reported in multiple studies^71−73^ with fibrils formed *in vitro* in high salt concentrations forming more twisted periodical fibrils being more toxic. In a disease context and on a molecular level, aSyn is likely to encounter higher Ca^2+^ concentrations in the extracellular space, especially due to neuronal damage or calcium dysregulation. Since aSyn can re-enter cells through endocytosis, it is conceivable that aSyn is consequently able to seed and template aggregation of endogenous aSyn, propagating the twisted fibril structures. A recent study on *ex vivo* samples from post-mortem tissue has found variations in periodicity across different clinical phases of PD.^74^ The fibrils have displayed distinct periodic patterns influenced by the clinical stage, with shorter and more twisted fibrils arising in later PD stages, further suggesting a correlation between aSyn propagation, the ionic environment, fibril structure, and disease progression.

Overall, our findings underscore the critical role of the ionic environment in regulating the structural dynamics and aggregation propensity of aSyn and indicate an effect of the presence of ions on fibril polymorphism. By revealing how specific ions influence aSyn behavior, this study contributes to a deeper understanding of the molecular underpinnings of PD and other synucleinopathies and opens new avenues for therapeutic intervention by targeting aSyn, in particular in the extracellular space, where calcium concentrations are higher before it can re-enter the neighboring neuron and enhance disease propagation.

## Methods

### Thioflavin-T (ThT) Binding Assay in 96-Well Plates

Thioflavin-T (ThT) kinetic assays are used to monitor the aggregation of aSyn in different buffer conditions. For sample preparation, 20 μM (final concentration) of freshly made ThT solution (Abcam, Cambridge, UK) in distilled water is added to 50 μL of 20 μM, aSyn in 20 mM Tris pH 7.4, supplemented with 2 mM CaCl_2_ (“Ca”), 150 mM NaCl (“Na”), 150 mM NaCl and 2 mM CaCl2 (“Na + Ca”), or 150 mM KCl (“K”).

All samples are loaded in nonbinding, clear 96-well plates (Greiner Bio-One GmbH, Germany), which are then sealed with a SILVERseal aluminum microplate sealer (Grenier Bio-One GmbH). Fluorescence measurements are taken using a FLUOstar Omega microplate reader (BMG LABTECH GmbH, Ortenbery, Germany). Excitation is set at 440 nm, and the ThT fluorescence intensity is measured at 480 nm emission with a 1300 gain setting. The plates are incubated with double orbital shaking for 300 s before the readings (every 60 min) at 300 rpm. Three repeats are performed with 6 replicates per condition. Each repeat is performed with a different purification batch of aSyn (biological replicate). Data are normalized to the well with the maximum fluorescence intensity for each plate, and the empirical aggregation parameters *t*_lag_*, t*_50_, and *k*, are calculated for each condition based on the equation
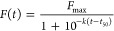
where *F* is the normalized fluorescence to the highest value recorded in the plate repeat, *F*_max_ is the maximum fluorescence at the plateau, *k* is the slope of the exponential phase of the curve, and *t*_50_ is the time when .

One-way ANOVA is used to calculate the statistical significance between samples using GraphPad Prism 8 (GraphPad Software).

### SEC-HPLC (Size Exclusion–High-Performance Liquid Chromatography)

At the end of the ThT-based aggregation assays, the amount of the remaining monomer of aSyn in each well is determined by analytical size exclusion chromatography with HPLC (SEC-HPLC). SEC analysis is performed on the Agilent 1260 Infinity HPLC system (Agilent Technologies, U.K.) equipped with an autosampler and a diode array detector using an AdvanceBio SEC column (Agilent Technologies, U.K.) in 20 mM Tris pH 7.4 at 0.8 mL/min flow-rate. 25 μL of each sample is injected onto the column, and the elution profile is monitored by UV absorption at 220 and 280 nm. The area under the peaks in the chromatogram of absorption at 280 nm is calculated to provide the monomer concentration. Samples for a standard curve spanning from 5 to 40 uM aSyn are run on the column to relate the area under the curve to protein concentration.

### 1H–^15^N HSQC NMR

NMR experiments are carried out at 10 °C on a Bruker spectrometer operating at a ^1^H frequency of 700 MHz equipped with triple resonance HCN cryo-probe, and data are collected using TopSpin 4.4.0 software (Bruker, AXS GmBH). The ^1^H–^15^N HSQC experiments are recorded using a data matrix consisting of 2048 (t2, 1H) × 220 (t1, 15N) complex points. Assignments of the resonances in ^1^H–^15^N HSQC spectra of aSyn are derived from our previous studies, and data are analyzed using Sparky 3.1 software. The perturbation of the ^1^H–^15^N HSQC resonances is analyzed using a weighting function in eq 1
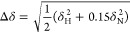
1

### Small-Angle Neutron Scattering (SANS)

Monomeric aSyn is isolated by gel filtration using a SuperdexTM 75 10/300 GL column (Cytivia, USA). The aSyn samples are buffer exchanged into D_2_O with 20 mM Tris pD 7.2 with the addition of either 150 mM NaCl, 2 mM CaCl_2_, or 150 mM NaCl + 2 mM CaCl_2_ using a HiTrapTM desalting Sephadex G-25 column (#29048684, Cytivia). A 200 uL portion of the sample solution is added to a 1 mm thick quartz cuvette (#QS, 100–1–40, Hellma, U.K.). Small-angle neutron scattering (SANS) experiments [https://dx.doi.org/10.5291/ILLDATA.8-03-974] are carried out on D22 at the Institut Laue-Langevin—The European Neutron Source, Grenoble, France. Samples are analyzed at three sample–detector distances (2.5, 5.6, and 17.6 m), with respective collimation lengths of 2.8m and 5.6 m. The wavelength is 6 Å ± 10%. The source aperture is 40 mm × 55 mm, and the beam aperture is 7 mm × 10 mm. Samples are maintained at 10 °C during the measurement. The raw counts from the detector for an empty cell and dark pattern are subtracted from the data sets. These are then scaled by the transmission and sample thickness, as well as the direct flux measurement, to obtain an absolute intensity. The data was reduced using Grasp.^75^ Data recorded in different configurations are buffer-subtracted and stitched together into individual 1D plots of *I*(*q*) *versusq* for each sample using specialized macros in Igor Pro.^76^

### Ensemble Optimization Method (EOM)

The experimental SANS data are analyzed using the ensemble optimization method (EOM)^77^ (https://www.embl-hamburg.de/biosaxs/eom.html). The WT aSyn sequence and the SANS experimental data are used without a defined structure, due to aSyn being intrinsically disordered, to predict 10,000 models in a RANdom Chains (RANCH) program. Genetic Algorithm Judging Optimization of Ensembles (GAJOE) is then used to select from 10,000 models, an ensemble of 50 models whose theoretical scattering intensity is most similar to the experimental scattering data. The pool of 10,000 models and the 50 most predictive models are presented as size distributions in the form of the radius of gyration (*R*_g_ (Å)).

### Hydrogen–Deuterium Exchange Mass Spectrometry (HDX-MS)

For labeling times ranging between 50 ms and 5 min, hydrogen–deuterium exchange (HDX) is performed using a fully automated, millisecond HDX labeling and online quench-flow instrument, ms2 min^55,56^ (Applied Photophysics, U.K.), connected to an HDX manager (Waters). For each cellular condition and three biological replicates, aSyn samples in the equilibrium buffer are delivered into the labeling mixer and diluted 20-fold with labeling buffer at 20̊C, initiating HDX. The duration of the HDX labeling depends on the mixing loops of varying length in the sample chamber of the ms2 min and the velocity of the carrier buffer, calibrated to a precision of 1 ms. The protein is labeled for a range of times from 50 ms to 5 min. Immediately postlabeling, the labeled sample is mixed with quench buffer in a 1:1 ratio in the quench mixer to arrest HDX. The sample is then centered on the HPLC injection loop of the ms2 min and sent to the HDX manager. For longer time points above 5 min, a CTC PAL sample handling robot (LEAP Technologies) is used. Protein samples are digested onto an enzymate immobilized pepsin column (Waters) to form peptides. The peptides are trapped on a VanGuard 2.1 mm × 5 mm ACQUITY BEH C18 column (Waters) for 3 min at 125 μL/min and separated on a 1 mm × 100 mm ACQUITY BEH 1.7 μm C18 column (Waters) with a 7 min linear gradient of acetonitrile (5–40%) supplemented with 0.1% formic acid. Peptide samples do not require the initial peptic digestion step. The eluted peptides are analyzed on a Synapt G2-Si mass spectrometer (Waters). An MSonly method with a low collisional activation energy is used for peptide-only HDX, and an MS/MS ETD fragmentation method is used for HDX-MS-ETD. Deuterium incorporation into the peptides and ETD fragments is measured in DynamX 3.0 (Waters).

### ETD Fragmentation of aSyn Peptides

The ETD reagent used is 4-nitrotoluene. The intensity of the ETD reagent per second, determined by the glow discharge settings, is tuned to give a signal of approximately 1 × 10^7^ counts per second (makeup gas flow: 35 mL/min, discharge current 65 μA) to give efficient ETD fragmentation. Instrument settings are as follows: sampling cone 30 V, trap cell pressure 5 × 10^–2^ mbar, trap wave height 0.25 V, trap wave velocity 300 m/s, transfer collision energy 8 V, and transfer cell pressure 8 × 10^–3^ mbar. Hydrogen–deuterium scrambling is measured using peptide precursor, as described in Phillips et al.^78^ under the same instrument conditions.

### HDX-MS Data Analysis

The raw data are processed, and assignments of isotopic distributions are reviewed in DynamX 3.0 (Waters). The postprocessing analysis is performed using HDfleX.^60^ Briefly, the back-exchange-corrected data points for each peptide and ETD fragment are fitted by using eq 2 in one-phase.
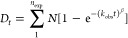
2

As the rate of HDX is affected by pH and ionic strength, which are not controlled in this study, it is crucial to normalize the solution effects among the different conditions being compared. Here, we use an empirical approach to normalization using the unstructured peptide bradykinin (RPPGFSPFR)^79,80^ to deconvolute the solution effects of the HDX from the protein structural changes. Due to the unstructured nature of bradykinin, all of the differences in deuterium uptake seen from the different buffers can be assumed to be strictly from the changes in the chemical exchange rate effects rather than from structural effects. HDX-MS experiments are performed on bradykinin under the exact same conditions as the protein of interest. Following back-exchange correction and curve-fitting, two fitted parameters, *k*_obs_ and β, are extracted. A 2D scaling factor is determined for each coordinate to transform it to the reference coordinate. This scaling factor, consisting of a value for *k*_obs_ and one for β, is multiplied to the fitted parameters for all of the buffer conditions. The empirical factor correction for each condition can then be applied to the uptake curves of the protein of interest, thereby normalizing the intrinsic exchange rate effects and allowing the structural effects to be clearly distinguished. The ETD fragments are combined with the peptide data using HDfleX^60^ to give the absolute uptake information across the entire protein.

### Statistical Significance Analysis

The hybrid significance testing method, along with data flattening, is used here and is described elsewhere.^60^

### Molecular Dynamics Simulations

Three aSyn models are generated from the PDB structures 2N0A, 8A9L, and 8ADS. Missing residues in 8A9L and 8ADS are supplemented by residues from 2N0A. For each initial structure, six simulations are run with differing ionic constituents. Preliminary simulations (not described) show approximately 25 calcium-binding sites per monomer. The intention of these simulations was to restrict, satiate, and saturate the calcium-binding sites, as such, simulations are carried out in the following conditions: “No Salt” contained the minimum ions required to equilibrate the system (10 Na^+^), “Unsaturated CaCl_2_” (20 Ca^2+^ ions), “CaCl_2_” (34 Ca^2+^ ions), “NaCl and CaCl_2_” (150 mM Na^+^ and 34 Ca^2+^ ions), “NaCl” (150 mM Na^+^), and “Saturated CaCl_2_” (150 mM Ca^2+^), in each instance, Cl^–^ ions were used to equilibrate the charge. Input files are prepared with Amber Tools^81^ packages tleap and parmed. Hydrogen atoms are added to pH 7. A truncated octahedron is solvated with Optimal Point Charges, OPC water[doi.org/10.1021/jz501780a], and ions appropriate for the relative investigation with a 14 Å gap from the protein to the periodic boundary. The system is parametrized using amber leaprc.protein.ff19SB, leaprc.water.OPC and the frcmod.ionslm_1264_opc[https://doi.org/10.1021/acs.jctc.0c00194]force fields hydrogen mass repartitioning is used to distribute heavy atom mass onto neighboring hydrogen atoms, allowing 4 fs time steps. Simulations are run using Amber22 on the Arc3 and Arc4 HPC at the University of Leeds, U.K. Backbone protein atoms are restrained during 5000 steps of minimization and 500 ps of equilibration. Production runs are conducted for an average of 1.9 μs (20NA), 1.6 μs (8A9L), and 2.7 μs (8ADS) at 303.15 K, and structures are written every 500 ps for analysis. A second set of fine time-scale simulations is performed using restart files from the above simulations at low RMSD areas of the trajectories for 3 ns at 303.15 K, and structures are written every 5 ps for analysis. The ion proximity frequency, water persistence times, and the distances between N and C-terminal and section center of masses are all calculated in PyMOL.^82^ The radius of gyration is calculated in cpptraj (Amber Tools) using the ‘radgyr’ function. The globularity of the protein was calculated as the ratio of the largest to the smallest eigenvalue (λ_max_/λ_min_) derived from the diagonalization of the covariance matrix of atomic positional fluctuations obtained using the “principal” function in CPPTRAJ, which performs principal component analysis on the trajectory. Density maps and graphs are prepared in Python using NumPy, Seaborn, Matplotlib, and SciPy.

### Atomic Force Microscopy (AFM) Analysis of Fibril Morphology

Fibrils formed at the end of ThT assays are analyzed by AFM. A freshly cleaved mica surface is coated with 0.1% poly l-lysine, washed with distilled H_2_O thrice, and dried under a stream of nitrogen gas. Samples from the microplate wells are then incubated for 30 min on the mica surface. The sample is washed thrice in the buffer of choice (for example, in 20 mM Tris, pH 7.4 for the Tris condition) to remove lose fibrils. Images are acquired in fluid using tapping mode on a BioScope Resolve AFM (Bruker) using ScanAsyst-Fluid + probes. 512 lines were acquired at a scan rate of 1.5 Hz per image with a field of view of 2–5 μm and for at least ten fields of view. Images are adjusted for contrast and exported from the NanoScope Analysis 8.2 software (Bruker). Measurements of fibril height and periodicity are performed by cross-sectioning across the fibril and across the fibril axis in NanoScope Analysis 8.2 software (Bruker). Statistical analysis of the height and periodicity measurements is performed in GraphPad Prism 8 (GraphPad Software).

## Data Availability

The authors declare that the data supporting the findings of this study are available in this paper and its Supporting Information files. Source data are provided with this paper. All mass spectrometry.raw files will be made available upon request to J.J.P.

## Supporting Information Available

Additional experimental details (HDX-MS technical details), additional and raw data: ThT assay individual traces, including aggregation in the presence of KCl, SANS traces, and EOM fitting distributions, HDX fittings and bradykinin-based corrections, HDX scrambling tests, HSQC NMR CSPs represented as histograms; MD simulations: metrics, 2D-RMSD, representative snapshots of the different starting structures, AFM images of aSyn fibrils, and height/periodicity data (PDF)
Source data for HDX and ThT experiments included in Supplementary Information (XLSX)

## Notes

**Code Availability Statement** This study uses in-house-developed software available to download: [http://hdl.handle.net/10871/127982].^60^

## Abbreviations

aSyn: α-synuclein
PD: Parkinson’s disease
NMR: nuclear magnetic resonance
HDX-MS: hydrogen–deuterium exchange mass spectrometry
SANS: small-angle neutron scattering
MD: molecular dynamics
IDP: intrinsically disordered protein
Rg: radius of gyration
SEC: size exclusion chromatography
ESI: electrospray ionization
TOF: time-of-flight
RMSD: root-mean-square deviation
DLS: dynamic light scattering
HSQC: heteronuclear single quantum coherence
CSP: chemical shift perturbationse
PDB: protein data bank
