# Supplementary material for: Local Ionic Conditions Modulate the Aggregation Propensity and Influence the Structural Polymorphism of α-Synuclein
Source: J Am Chem Soc. 2025 Apr 10;147(16):13131–45. doi: 10.1021/jacs.4c13473 (PMC12023029; doi:10.1021/jacs.4c13473)
Supplement: Supplementary file 1 — ja4c13473_si_001.pdf [file ja4c13473_si_001.pdf]

Supplementary Information

Local ionic conditions modulate the aggregation propensity and influence the structural polymorphism of alpha-synuclein

Maria Zacharopoulou[1], Neeleema Seetaloo[2], James Ross[3], Amberley D. Stephens[1], Giuliana Fusco[4,5], Thomas M. McCoy[1], Wenyue Dai[1], Ioanna Mela[1], Ana Fernandez-Villegas[1], Anne Martel[6], Alexander F. Routh[1], Alfonso De Simone[4,5], Jonathan J. Phillips[2], Gabriele S. Kaminski Schierle[1]*

[1] Department of Chemical Engineering and Biotechnology, University of Cambridge, Philippa Fawcett Drive, Cambridge CB3 0AS, UK

[2] Living Systems Institute, University of Exeter, Stocker Road, Exeter EX4 4QD, UK

[3] School of Molecular and Cellular Biology and Astbury Centre for Structural Molecular Biology, University of Leeds, Leeds LS2 9JT, UK

[4] Department of Chemistry, University of Cambridge, Cambridge CB2 1EW, UK.

[5] Department of Life Sciences, Imperial College London, London SW7 2AZ, UK.

[6] Institut Laue Langevin, 71 avenue des Martyrs, Grenoble CS 20156, 38042, France

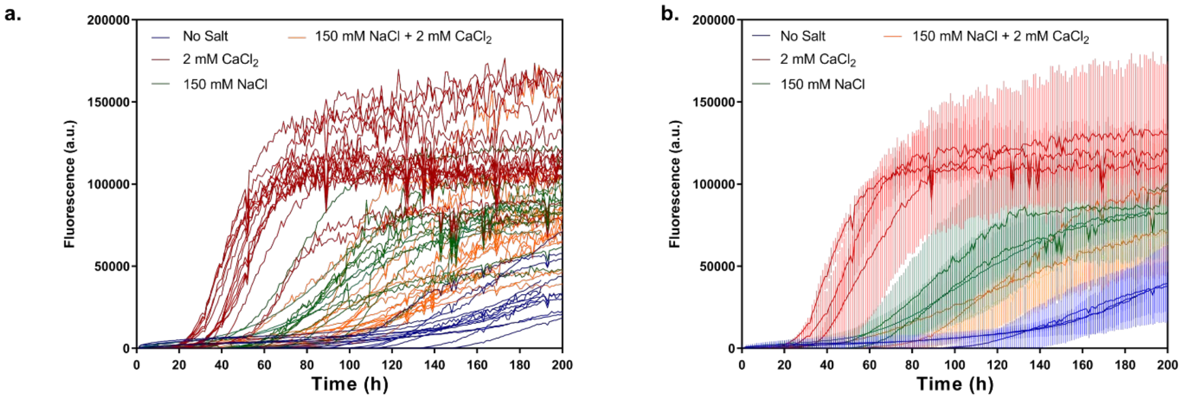

Figure S1: (a) All individual ThT aggregation kinetics replicates across biological repeats and (b) average of traces for each biological replicate (N=3). Fluorescence intensity plotted in arbitrary units (a.u.).

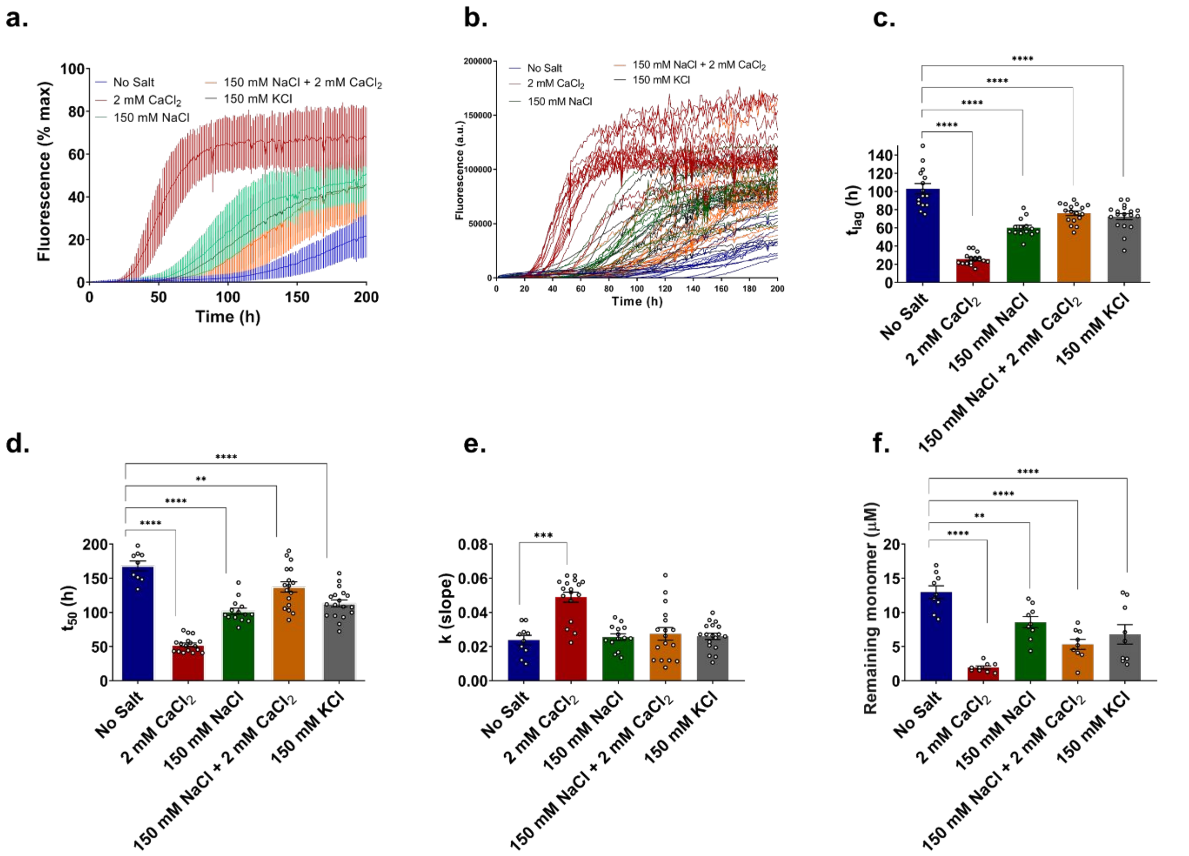

Figure S2: ThT aggregation kinetics of WT aSyn is increased in the presence of all ions (Na^+, Ca^{2+}, K^+). The conditions plotted here are aSyn in 20 mM Tris pH 7.4, with addition of 2 mM $CaCl_2$, 150 mM NaCl, 150 mM NaCl and 2 mM $CaCl_2$, 150 mM KCl. At least 6 replicates across three biological repeats were collected per condition. **a.** Kinetic traces. The average between traces of the same

condition is shown in the graph and errors indicate 1 s.d., **b.** Each individual kinetic trace. Fluorescence intensity plotted in arbitrary units (a.u.) **c**. lag time (t_{lag}), **d**. time to reach 50 % of maximum aggregation (t_{50}) and **e.** the slope of the curve *k* were calculated by fitting Equation 1, and the mean plus error (1 s.d.) are displayed in the graphs. **f.** Remaining monomer concentration (μM) at the end of the aggregation assay was determined using SEC-HPLC. An ordinary ANOVA was used to calculate statistical significance between samples and significant differences are reported on the graph with an asterisk *.

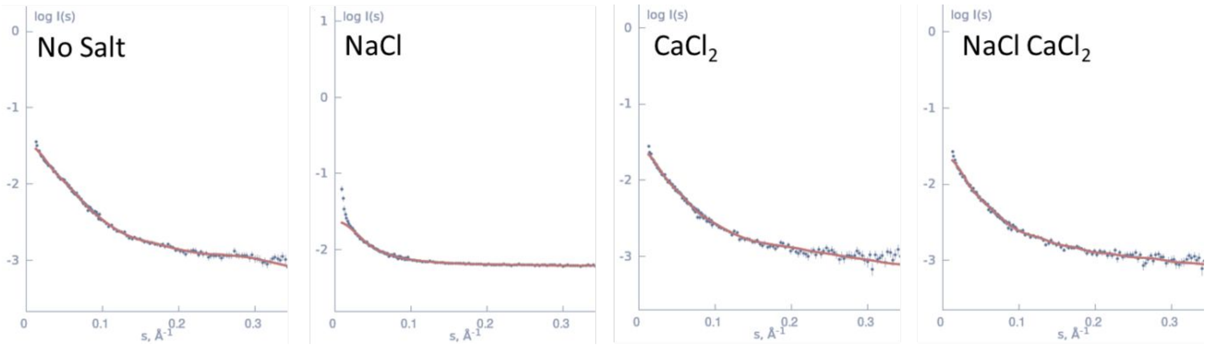

Figure S3: SANS of aSyn in different ionic conditions. GAJOE was used to fit to the best ensemble prediction from the data (red fitted line)

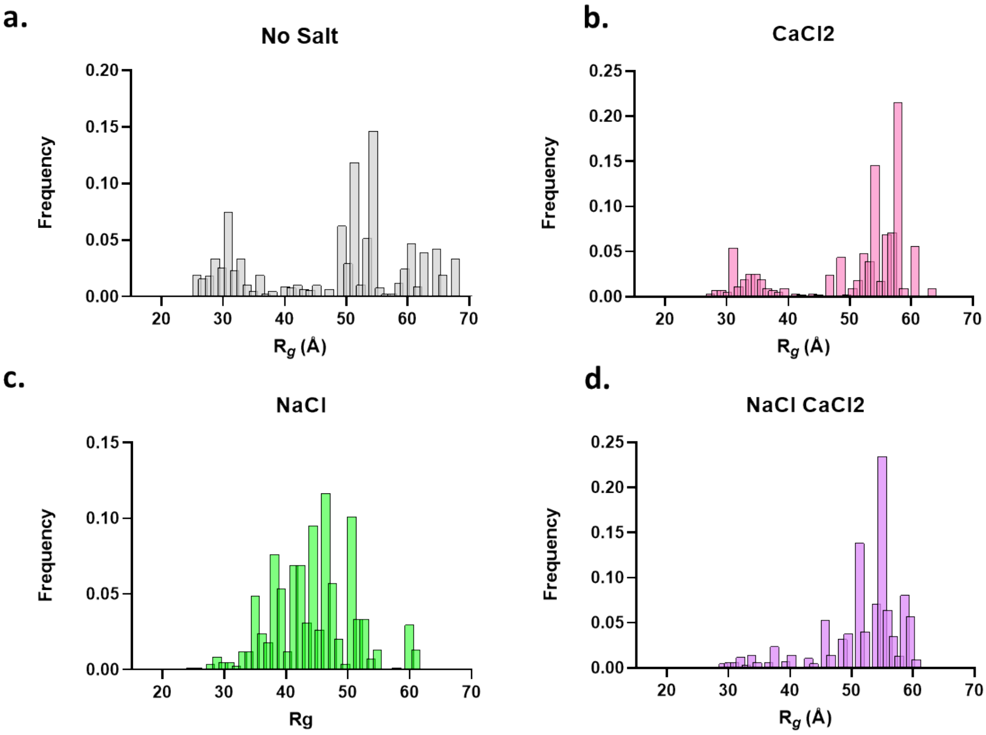

Figure S4: Ensemble optimisation method (EOM) of SANS data shows varying distribution of the radius of gyration of aSyn in different ionic conditions. A pool of 1000 independent models based upon sequence and structural information (i.e., no defined structure for an IDP) is generated.

The predicted scattering intensity from the models is compared to the experimental data and the 50 models of the best fit to the experimental data are selected as the most accurate representations, represented in the graphs.

Table S1: HDX-MS experimental technical details.

**Data Set**	**aSyn**
HDX reaction details	20˚C, pH 7.4<br>20 mM Tris<br>20 mM Tris + 2 mM $CaCl_2$<br>20 mM Tris + 150 mM NaCl<br>20 mM Tris + 150 mM NaCl + 2 mM $CaCl_2$
HDX time course (ms)	50, 100, 250, 500, 1000, 10000, 30000, 300000.
Back-exchange (mean / IQR)	39.3% / 15.1%
Number of Peptides	30
Sequence coverage	100%
Peptide Redundancy	3.87
Replicates (biological or technical)	1 (biological), 3 (technical)
Significant differences in HDX (delta HDX > X Da)	0.35 (95% CI, quartiles method of outlier removal)

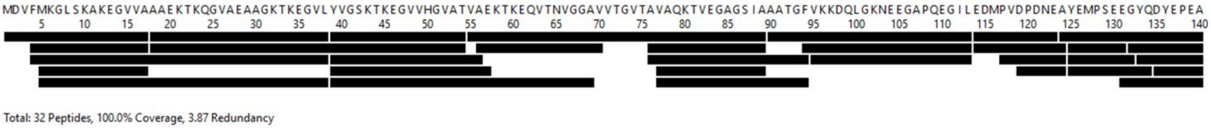

Figure S5: Peptide coverage map in HDX-MS experiments. A total of 30 peptides were assigned with 100% aSyn sequence coverage and 3.87 degree of redundancy.

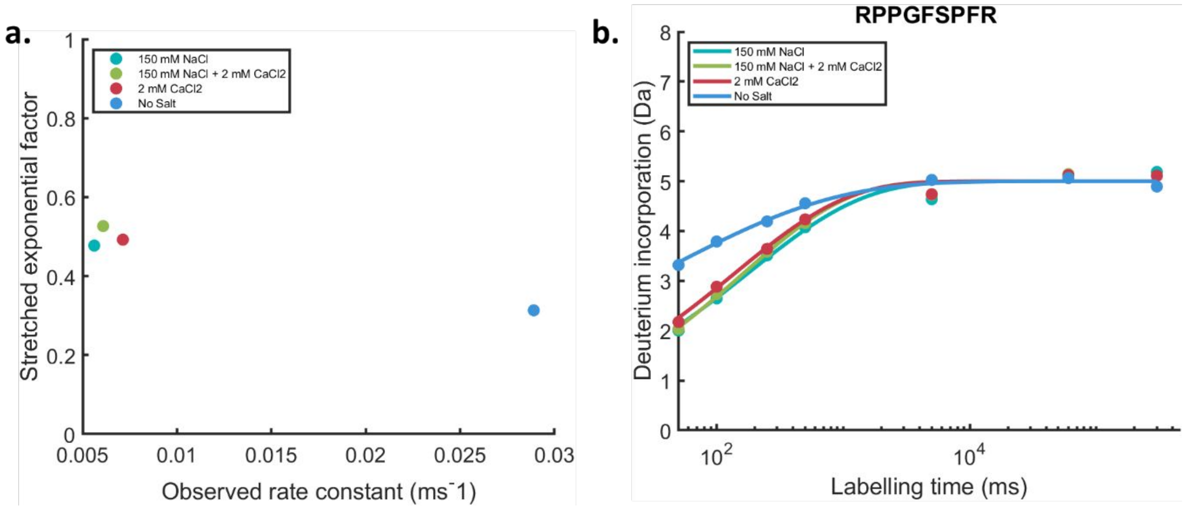

Figure S6: Unstructured peptide bradykinin for the calibration of chemical exchange rate effects; **a.** 2D plot of extracted fitted parameters k_{obs} and β, with the “No Salt” condition (blue dot) chosen as

reference state; **b.** Uptake curve for bradykinin in the four conditions; Data points are the mean of n=3 technical replicates.

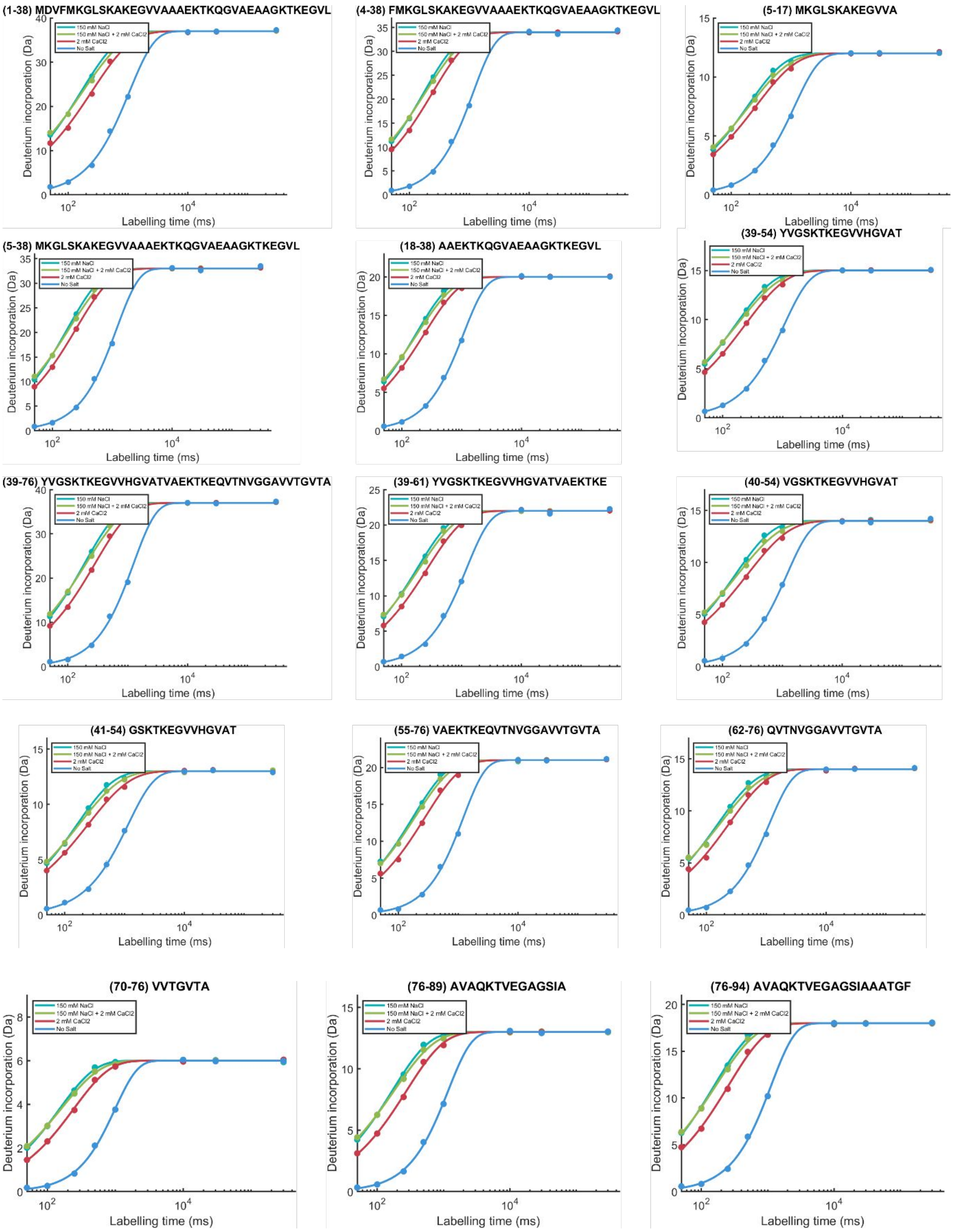
(1-38) MDVFMKGLSKAKEGVVAAAEKTKQGVAEAAGKTKEGVL
(4-38) FMKGLSKAKEGVVAAAEKTKQGVAEAAGKTKEGVL
(5-17) MKGLSKAKEGVVA
(5-38) MKGLSKAKEGVVAAAEKTKQGVAEAAGKTKEGVL
(18-38) AAEKTKQGVAEAAGKTKEGVL
(39-54) YVGSKTKEGVVHGVAT
(39-76) YVGSKTKEGVVHGVATVAEKTKEQVTNVGGAVVTGVTA
(39-61) YVGSKTKEGVVHGVATVAEKTKE
(40-54) VGSKTKEGVVHGVAT
(41-54) GSKTKEGVVHGVAT
(55-76) VAEKTKEQVTNVGGAVVTGVTA
(62-76) QVTNVGGAVVTGVTA
(70-76) VVTGVTA
(76-89) AVAQKTVEGAGSIA
(76-94) AVAQKTVEGAGSIAAATGF
150 mM NaCl
150 mM NaCl + 2 mM CaCl2
2 mM CaCl2
No Salt
Deuterium incorporation (Da)
Labelling time (ms)

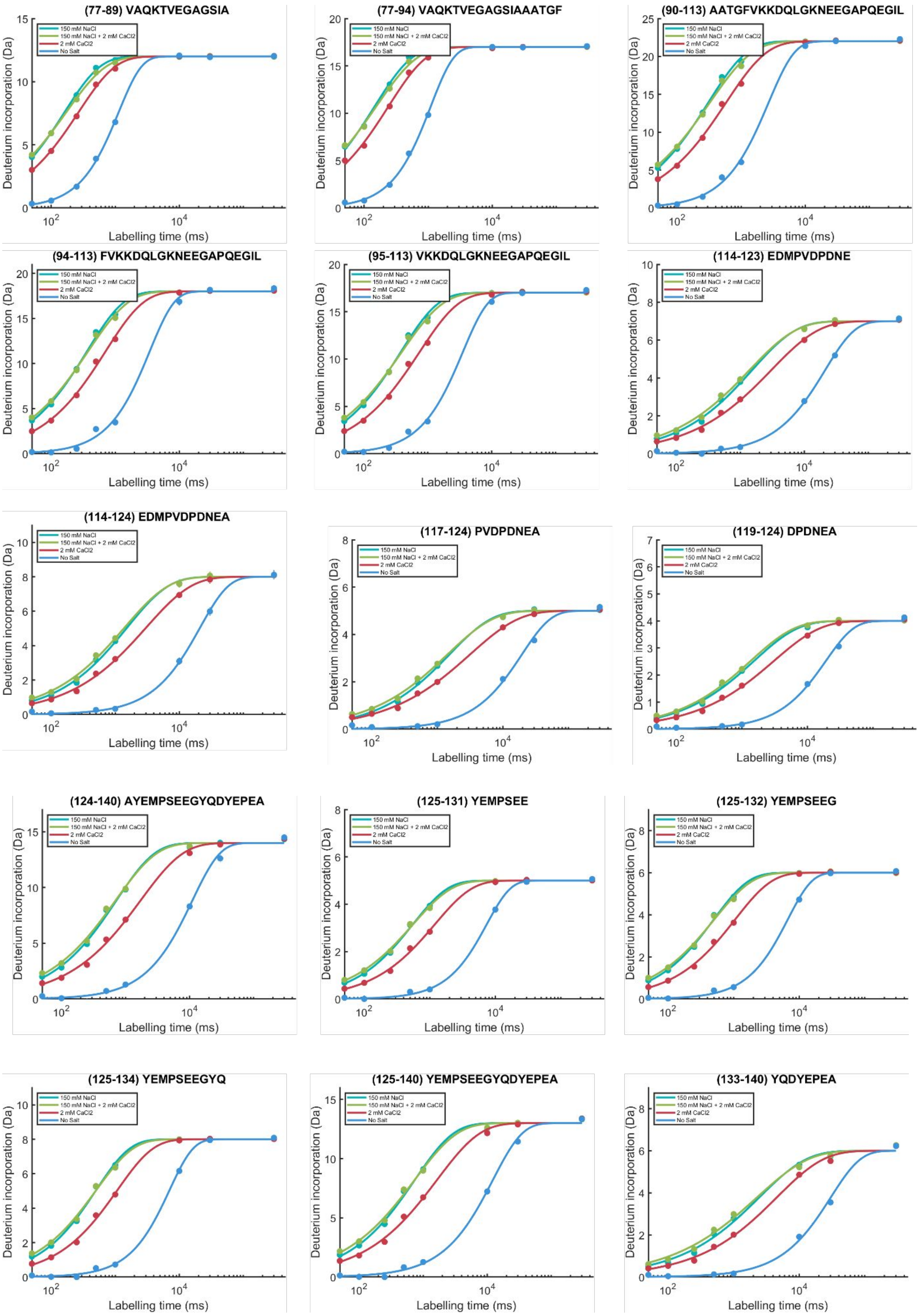

Figure S7: Empirically adjusted deuterium uptake plots for aSyn equilibrated in No Salt, 2 mM $CaCl_2$, 150 mM NaCl, and 150 mM NaCl and 2mM $CaCl_2$ conditions. Timepoints collected: 50 ms,

100 ms, 250 ms, 500 ms, 1000 ms, 10 s, 30 s, 300 s. Y-axis shows deuterium incorporation in Da and x-axis shows the log scale labelling times in ms. Data points are the mean of n=3 technical replicates.

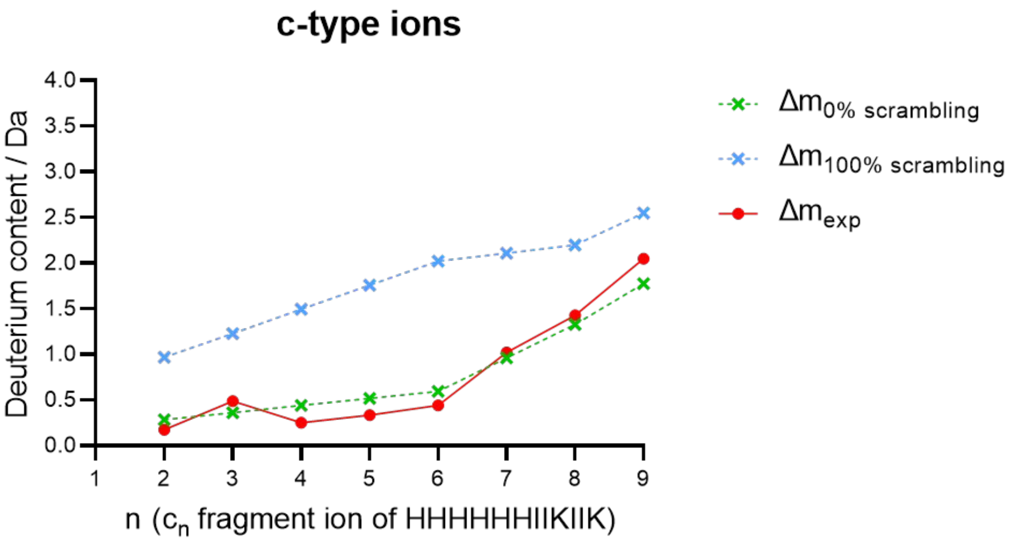

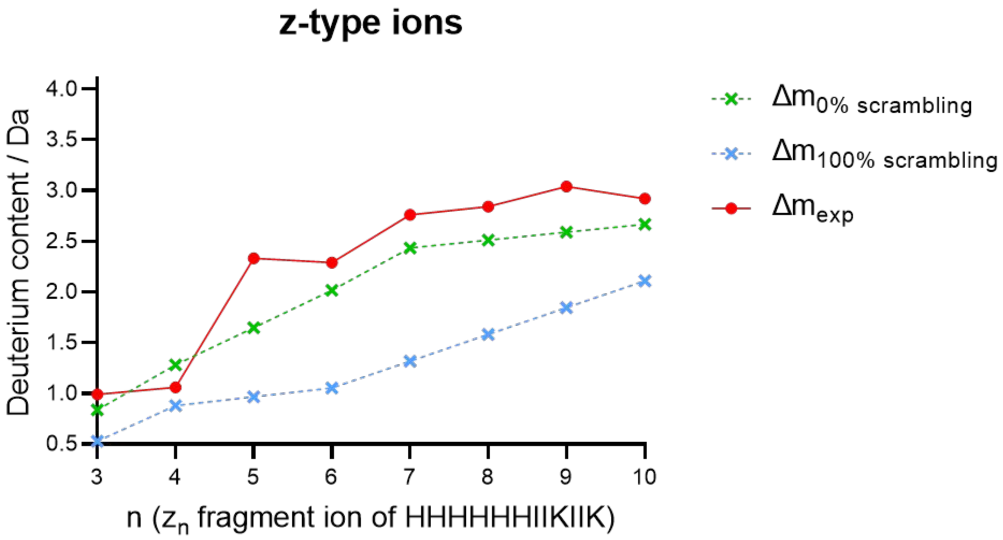

Figure S8: Hydrogen-deuterium scrambling is not observed in the c and z fragments of peptide P1 under identical conditions as aSyn experiments. The green and blue dotted lines on the plots represent the 0% and 100% theoretical scrambling data for this peptide respectively, and the red line shows the experimental data. From the plots, it can be inferred that the conditions chosen for ETD fragmentation do not cause H/D scrambling as the experimental data line (red) approaches the 0% scrambling line (green) for both types of fragments.

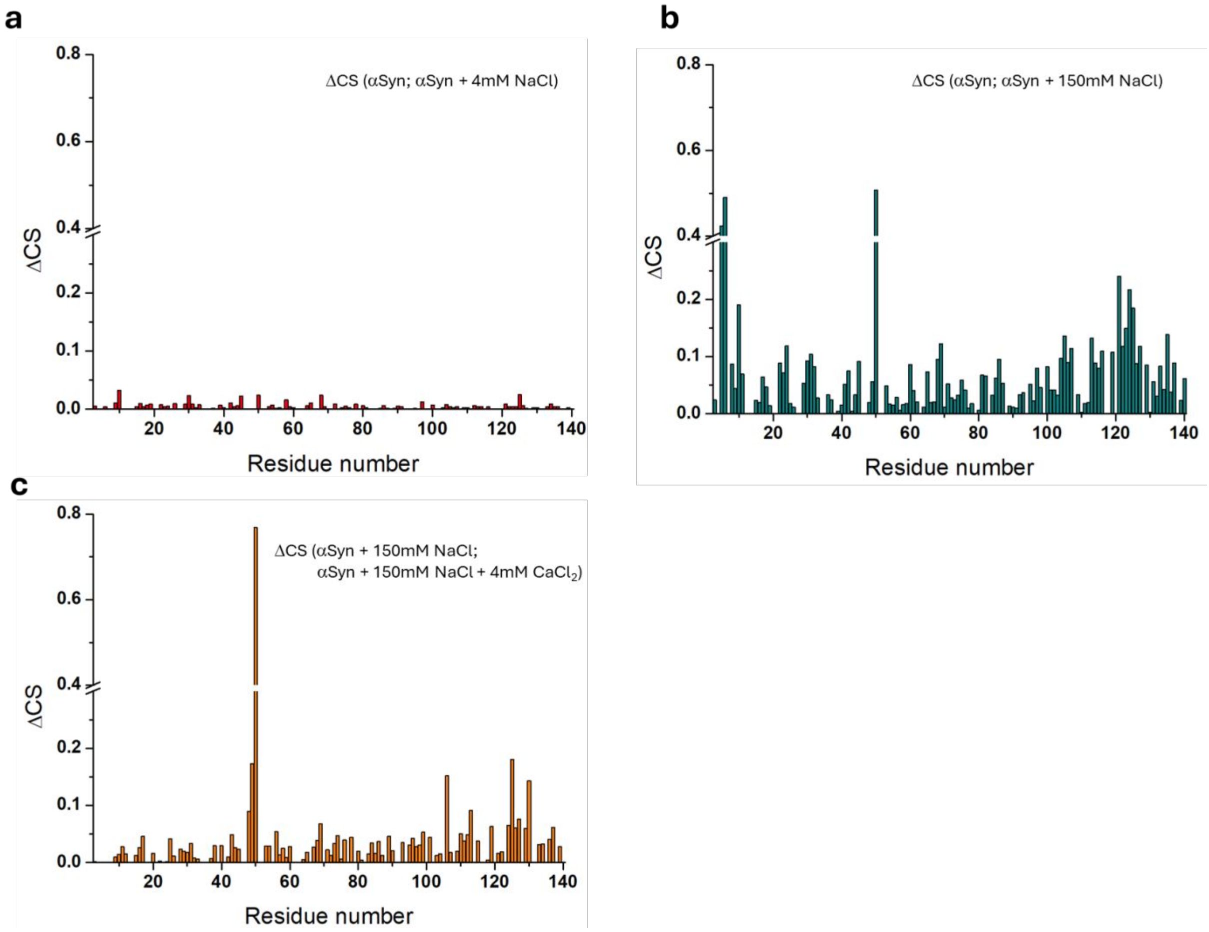

Figure S9: Chemical Shift Perturbations in the NMR experiments, represented as histograms (corresponding to Figure 2). The ΔCS were calculated using the following formula: $\Delta CS = \sqrt{\frac{(\delta_H^2 + 0.15\delta_N^2)}{2}}$

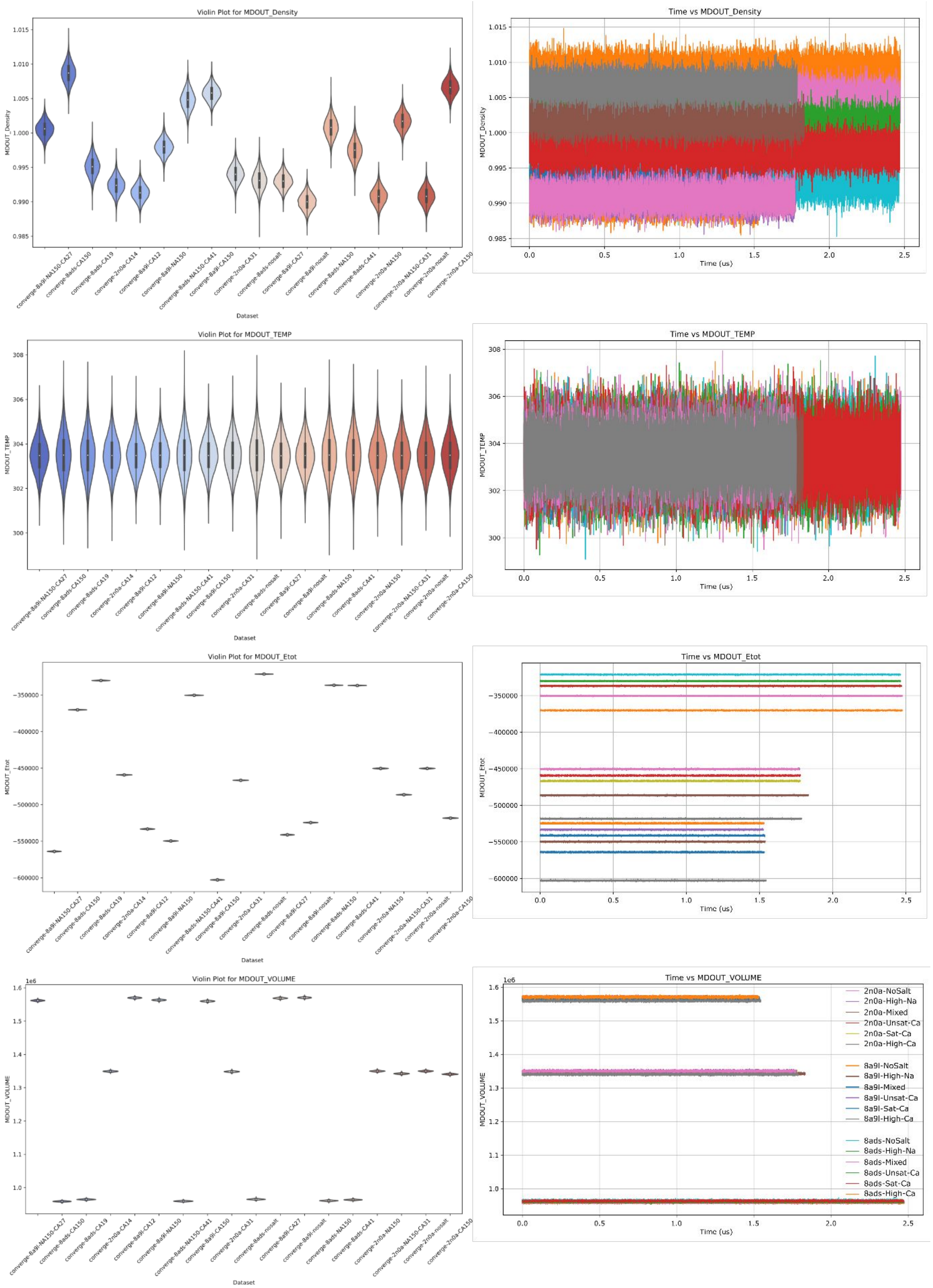

Figure S10: Metrics for the MD simulations, showing density, temperature, total energy and volume as both violin plots and against time for each simulation.

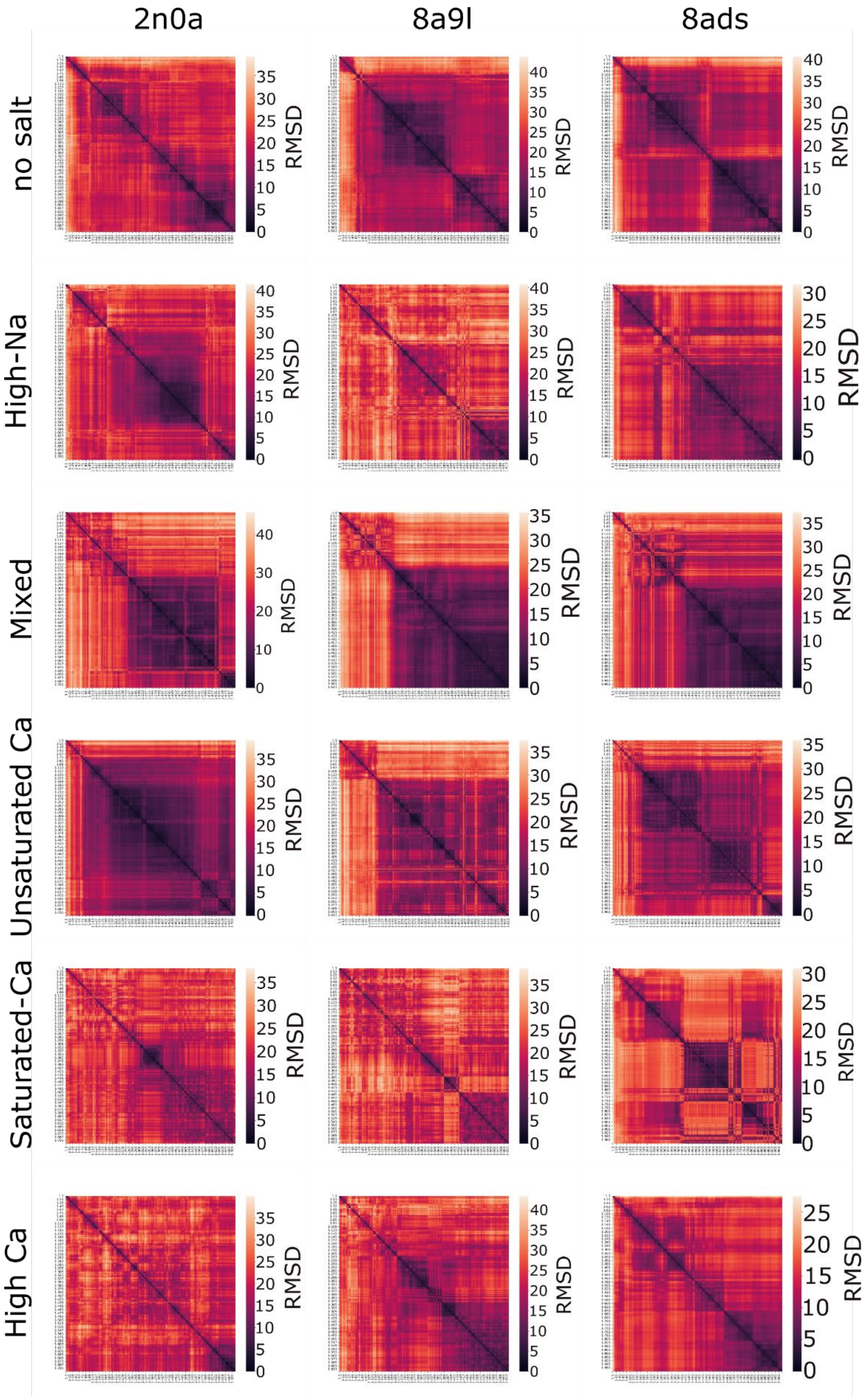

Figure S11: 2D-RMSD of all simulations. 2D-RMSD show the deviation between each frame (x-axis) and each other frame (y-axis) as a heat map. In these simulations we can observe a variety of features, such as simulations which find a low-energy well (8a9l-mixed), those moving to new states (8a9l-nosalt) and those transitioning between previously occupied conformations (8ads-saturated-Ca).

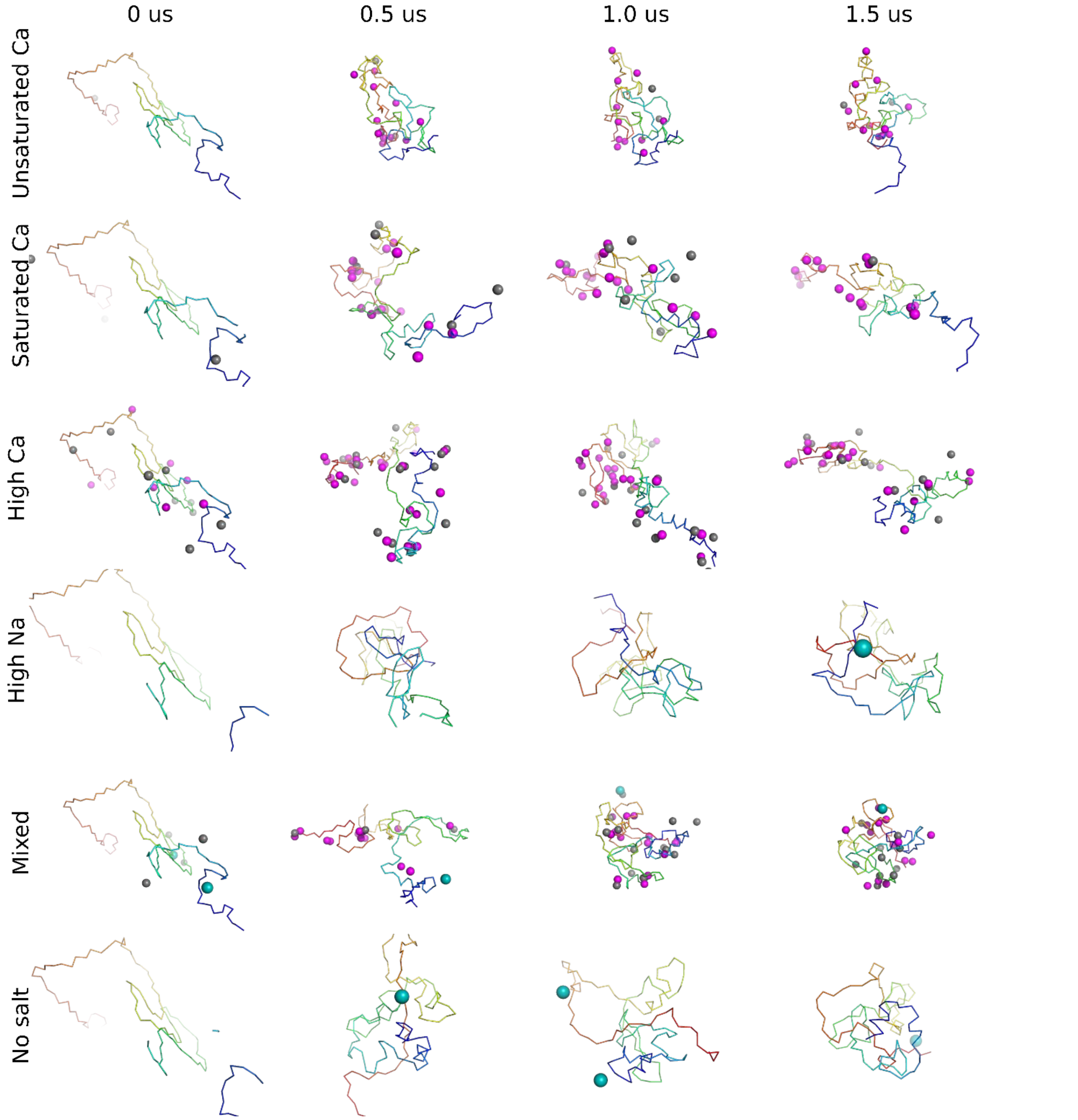

Figure S12: MD simulation snapshots from 2n0a starting structure. Protein coloured from N-terminus (blue) to C-terminus (red), ions shown within 4Å of protein, Cl^- ions in grey, Na^+ ions in cyan, Ca^{2+} ions in magenta.

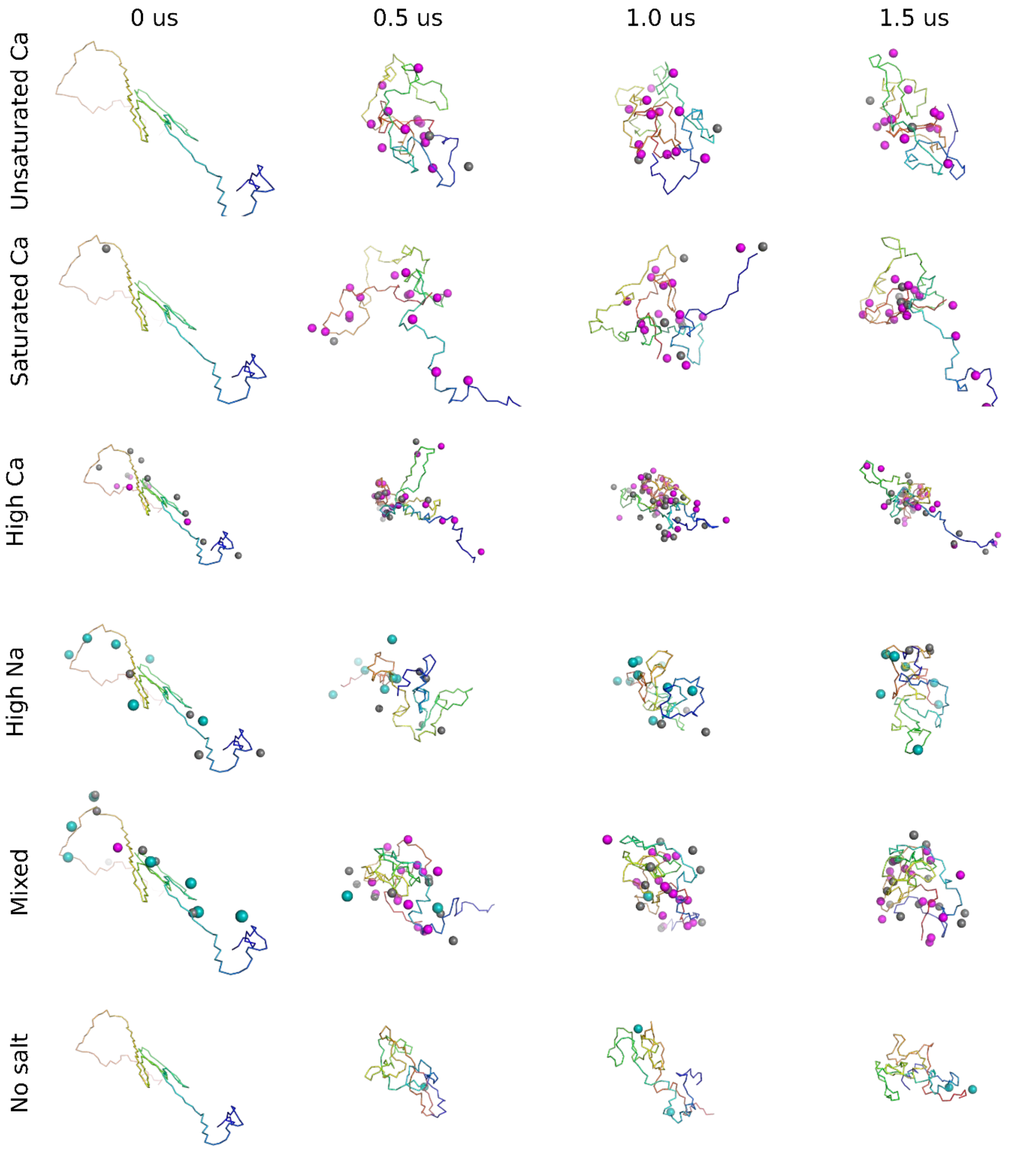

Figure S13: MD simulation snapshots from 8a9l starting structure: Protein coloured from N-terminus (blue) to C-terminus (red), ions shown within 4Å of protein, Cl^- ions in grey, Na^+ ions in cyan, Ca^{2+} ions in magenta.

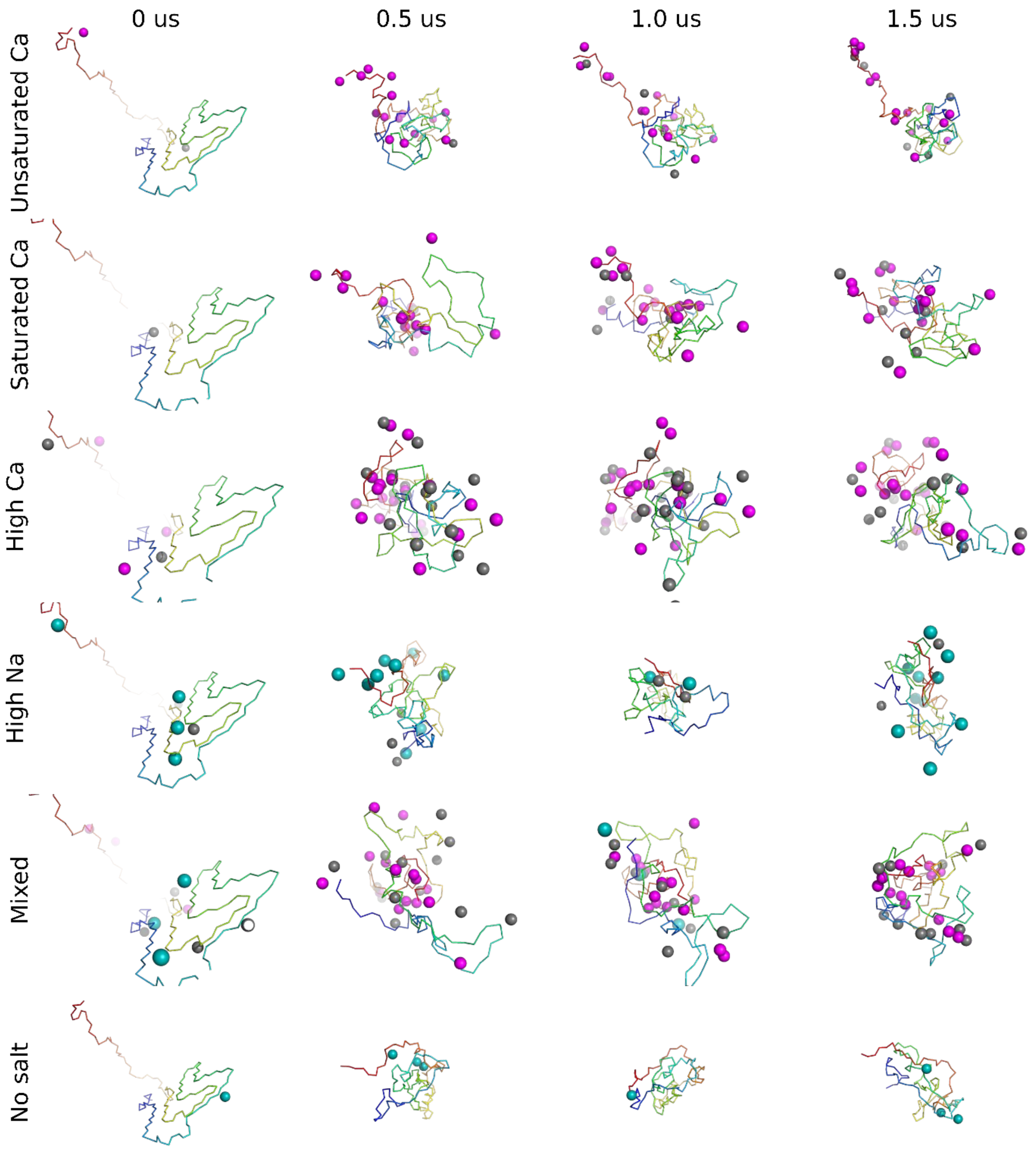

Figure S14: MD simulation snapshots from 8ads starting structure: Protein coloured from N-terminus (blue) to C-terminus (red), ions shown within 4Å of protein, Cl^- ions in grey, Na^+ ions in cyan, Ca^{2+} ions in magenta.

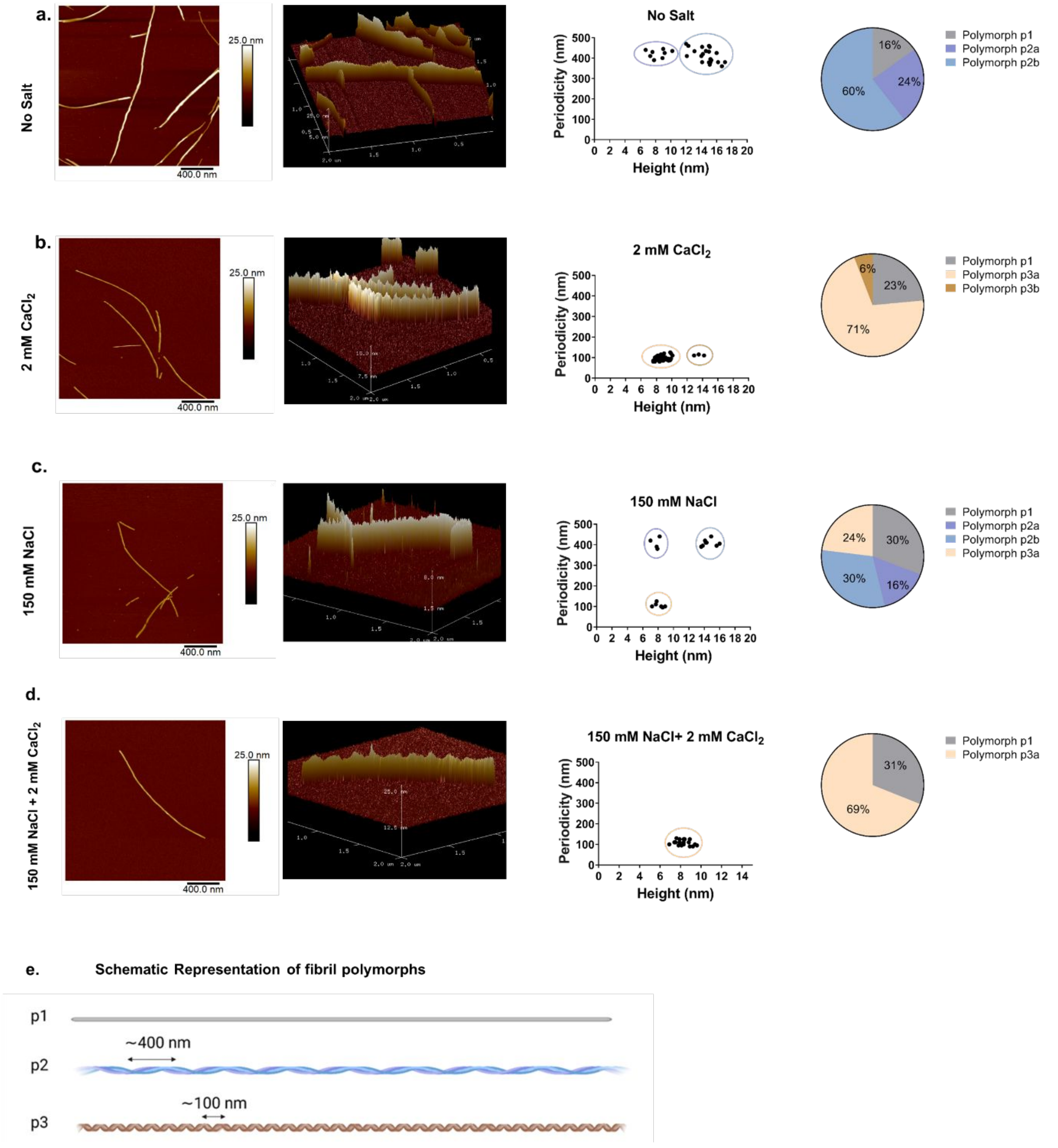

Figure S15: Different ionic conditions bias the fibril polymorphism of aSyn, with Ca^{2+} inducing more twisted fibril structures as shown by AFM. a.-d.: Fibrils formed in no salt conditions, with 2 mM $CaCl_2$, 150 mM NaCl, 150 mM NaCl and 2 mM $CaCl_2$**,** respectively. First column: representative AFM image of the sample. Second column: 3D representation of the same image, Third column: Scatter plots of fibril periodicity and height. The heights of the fibrillar species were obtained by averaging measurements from individual fibrils (~ 4 points per individual fibril). Fibril periodicity was determined by sectioning along the length of a fibril and averaging the distance measured between two adjacent peaks in height. The different

polymorph populations are indicated with circles on the scatter plots. Polymorph p1 is not plotted on the scatter plot as it has no periodicity. Fourth column: Relative abundance (%) of each fibril polymorph in the sample. **e.**: Schematic representation of the three fibril polymorph populations identified across the samples. AFM images were collected in ScanAsyst Fluid tapping mode, with a resolution of 512 lines with a scan rate of 1.5 Hz. Fibril analysis was performed using NanoScope Analysis v1.9 software**.**
